# m6A RNA methylation regulates the transcription factors JUN and JUNB in TGF-β-induced epithelial–mesenchymal transition of lung cancer cells

**DOI:** 10.1016/j.jbc.2022.102554

**Published:** 2022-09-29

**Authors:** Kusuma Suphakhong, Minoru Terashima, Sasithorn Wanna-udom, Risa Takatsuka, Akihiko Ishimura, Takahisa Takino, Takeshi Suzuki

**Affiliations:** 1 Division of Functional Genomics, Cancer Research Institute, Kanazawa University, Kanazawa, Ishikawa, Japan; 2 Division of Education for Global Standard, Institute of Liberal Arts and Science, Kanazawa University, Kanazawa, Ishikawa, Japan

**Keywords:** cancer biology, epithelial–mesenchymal transition, RNA methylation, gene expression, translation regulation, mRNA stability, m6A reader protein, cDNA, complementary DNA, CDS, coding sequence, EMT, epithelial–mesenchymal transition, HA, hemagglutinin, IgG, immunoglobulin G, m6A, N6-methyladenosine, m6A RNA-IP, m6A RNA immunoprecipitation, METTL, methyltransferase-like, RIP, RNA immunoprecipitation, QRT–PCR, quantitative RT–PCR, TF, transcription factor, TGF-β, transforming growth factor-beta

## Abstract

N6-methyladenosine (m6A) is the most common internal chemical modification of mRNAs involved in many pathological processes including various cancers. In this study, we investigated the m6A-dependent regulation of JUN and JUNB transcription factors (TFs) during transforming growth factor-beta–induced epithelial–mesenchymal transition (EMT) of A549 and LC2/ad lung cancer cell lines, as the function and regulation of these TFs within this process remains to be clarified. We found that JUN and JUNB played an important and nonredundant role in the EMT-inducing gene expression program by regulating different mesenchymal genes and that their expressions were controlled by methyltransferase-like 3 (METTL3) m6A methyltransferase. METTL3–mediated regulation of JUN expression is associated with the translation process of JUN protein but not with the stability of JUN protein or mRNA, which is in contrast with the result of m6A-mediated regulation of *JUNB* mRNA stability. We identified the specific m6A motifs responsible for the regulation of JUN and JUNB in EMT within 3′UTR of *JUN* and *JUNB*. Furthermore, we discovered that different m6A reader proteins interacted with *JUN* and *JUNB* mRNA and controlled m6A-dependent expression of JUN protein and *JUNB* mRNA. These results demonstrate that the different modes of m6A-mediated regulation of JUN and JUNB TFs provide critical input in the gene regulatory network during transforming growth factor-beta–induced EMT of lung cancer cells.

Epithelial–mesenchymal transition (EMT) is one of the crucial mechanisms causing cancer malignancies, such as invasion, metastasis, and resistance to therapy (1, 2). During EMT, tumor cells lose epithelial characters such as cell polarity and cell contacts and acquire invasive stem cell–like properties that expand their ability for local invasion and metastasis. A major inducer of EMT is transforming growth factor-beta (TGF-β) along with the cytokines and growth factors secreted by the tumor microenvironment. EMT is characterized by the dynamic and reversible changes in epithelial and mesenchymal gene expression (3). Epithelial cell markers such as E-cadherin and claudins are downregulated, whereas mesenchymal markers including vimentin, fibronectin, and N-cadherin are upregulated during EMT. Many transcription factors (TFs) are involved in the transcriptional regulation of EMT-related genes. Especially, TFs such as ZEB family, SNAIL family, and TWIST can activate EMT through the transcriptional repression of E-cadherin. Recently, nonredundant functions of EMT-related TFs have been emphasized (4). Because of the differential expression patterns in different tumor types, EMT-related TFs would have different functions and different target genes in a context-dependent manner. Therefore, careful analyses and discussions are important to understand their roles in various cancers. In addition, epigenetic regulations are considered as one of the critical mechanisms for EMT owing to its phenotypic plasticity (5, 6). Thus, we have investigated and found the essential roles of histone methylation, histone ubiquitination, and long noncoding RNAs in the transcriptional regulatory network during EMT (7, 8, 9, 10).

N6-methyladenosine (m6A) is the most prevalent internal chemical modification of mRNAs and long noncoding RNAs in eukaryotes and is implicated in alternative polyadenylation, pre-mRNA splicing, mRNA stability, and translation efficiency (11). This modification is controlled by a series of proteins identified as “Writer,” “Eraser,” and “Reader.” Methyltransferase-like 3 (METTL3), METTL14, WTAP, and RBM15 constitute the m6A methyltransferase complex, “Writer,” to catalyze m6A methylation. On the other hand, FTO and ALKBH5 function as “Eraser” to remove the *N*-methyl of m6A site, which maintains a dynamic nature of this modification (12). “Reader” includes the YTHDF family, IGF2BP family, and YTHDC family to transmit the modification signal. It is emerging that m6A modification plays an important role in diverse biological processes, including development, metabolism, stemness maintenance, and differentiation (13). Recent studies have also revealed that m6A regulation is involved in the development and progression of various types of cancer (14, 15).

It has been demonstrated that JUN family of TFs, JUN, JUNB, and JUND, are activated by various external stimuli and are involved in cellular proliferation, differentiation, and tumorigenesis (16). In particular, with respect to EMT regulation, it was reported that silencing of JUN enhanced E-cadherin expression and repressed N-cadherin in nasopharyngeal carcinoma cells (17) and suppressed SNAI2 expression in breast cancer cells (18). During transforming growth factor-beta (TGF-β)–induced EMT in mouse mammary epithelial cell line NMuMG, JUNB controls the induction of profibrotic factors such as fibronectin and tropomyosin, which are essential for cell–matrix adhesion and actin stress fiber formation (19). JUND was shown to be involved in arecoline-induced EMT of head and neck squamous cell carcinoma through the downregulation of ZO-1 (20). These studies suggested that the involvement of each JUN family member in EMT and the target genes it controls might be different in various cancer types. Previously, we have shown that JUNB is one of the important TFs regulated by METTL3 m6A methyltransferase during TGF-β-induced EMT of A549 and LC2/ad lung cancer cell lines (21). METTL3 influences the stability of *JUNB* mRNA through m6A methylation. However, the function of each JUN family member and its regulation by m6A modification during TGF-β-induced EMT in lung cancer cells are still largely unknown.

In this study, we tried to elucidate the function and regulatory mechanism of JUN family TFs in the expression of epithelial and mesenchymal genes during TGF-β-induced EMT of lung cancer cell. Our data indicated that JUN and JUNB but not JUND contributed to the EMT-inducing gene expression program by regulating different mesenchymal marker genes. We also found that m6A RNA modification by METTL3 regulated the expression of JUN at the protein level but controlled JUNB expression at the RNA level during EMT, which was mediated by the different m6A reader proteins.

## Results

### JUN and JUNB play an important role in the gene expression program during TGF-β-induced EMT process of A549 and LC2/ad lung cancer cells

**Figure 1:**
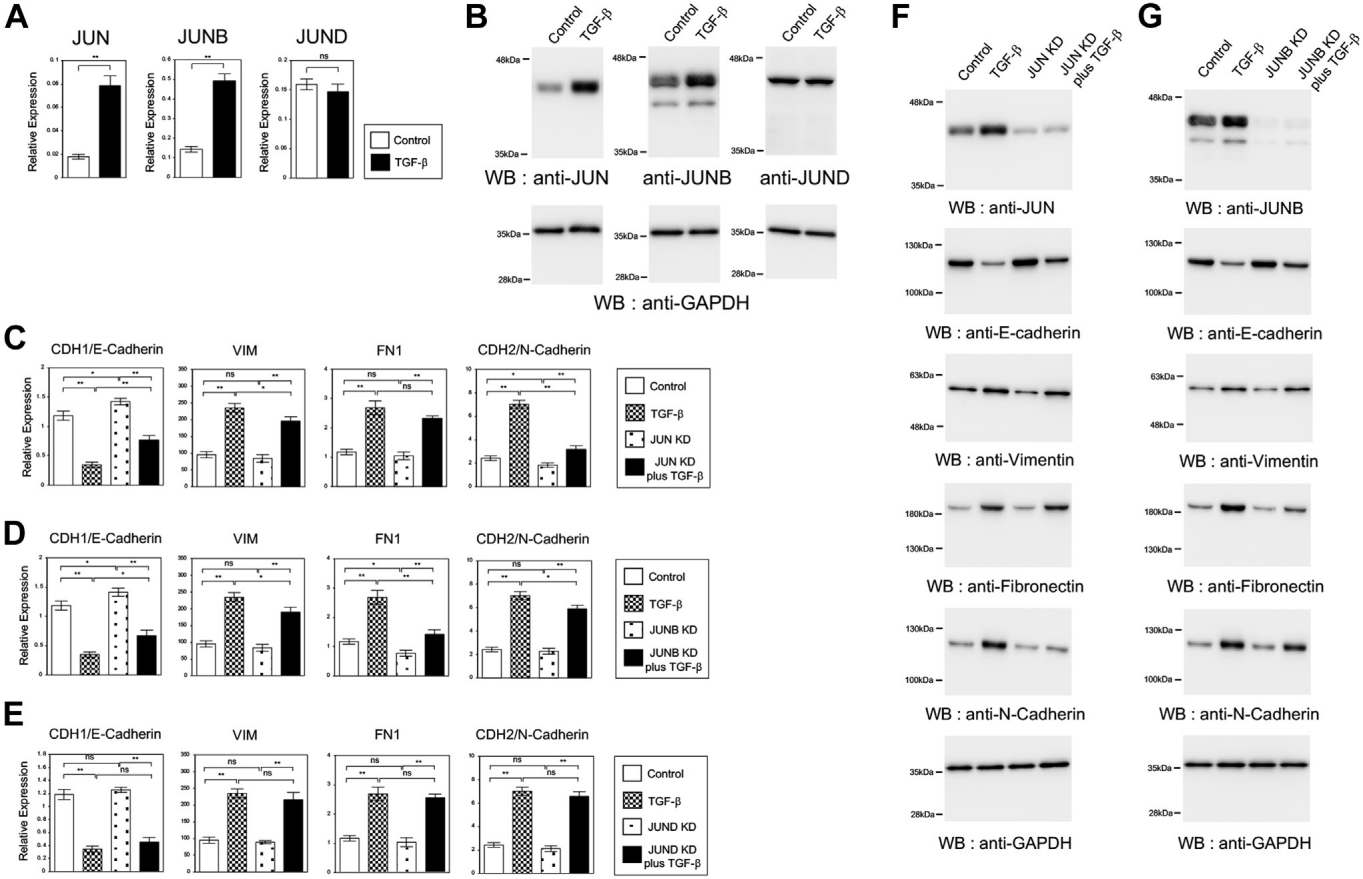
**Involvement of JUN and JUNB transcription factors in the expression of epithelial and mesenchymal marker genes during TGF-β-induced EMT of A549 lung cancer cells.***A*, the expression of *JUN*, *JUNB*, and *JUND* mRNAs in TGF-β-induced EMT of A549 cells. QRT–PCR was performed in A549 cells with or without TGF-β treatment (∗∗*p* < 0.01; ns, not significant). *B*, the expression levels of JUN family proteins in A549 cells. Immunoblotting was performed to detect JUN, JUNB, and JUND proteins in the cells with or without TGF-β treatment. As a control, anti-GAPDH antibody was used to show that equal amounts of proteins were loaded. *C*–*E*, the expression of EMT-related marker genes in JUN family knockdown A549 cells. QRT–PCR was performed to detect the expression of *CDH1*, *VIM*, *FN1*, and *CDH2* in A549 cells infected with lentivirus-expressing control shRNA or *JUN* shRNA#1 without or with TGF-β for 24 h (*C*) (n = 3) (∗∗*p* < 0.01; ∗*p* < 0.05; ns, not significant). The similar QRT–PCR results were shown for the knockdown of *JUNB* (*D*) and *JUND* (*E*) in A549 cells. *F* and *G*, knockdown of *JUN* and *JUNB* affected the protein expression changes of EMT-related marker genes induced by TGF-β in A549 cells. Immunoblotting of JUN (*F*), JUNB (*G*), E-cadherin, vimentin, fibronectin, N-cadherin, and GAPDH proteins was performed using the corresponding antibodies in A549 cells with the knockdown by *JUN* shRNA#1 (*F*) and *JUNB* shRNA#1 (*G*). EMT, epithelial–mesenchymal transition; QRT–PCR, quantitative RT–PCR; TGF-β, transforming growth factor-beta.

To validate the involvement of JUN family TFs in TGF-β-induced EMT of lung cancer cells, we first examined the changes in expression of *JUN*, *JUNB*, and *JUND* by TGF-β treatment (Figs. 1, *A* and *B* and S1). Quantitative RT–PCR (QRT–PCR) revealed that the expressions of *JUN* and *JUNB* but not of *JUND* mRNA were increased by TGF-β in A549 (Fig. 1*A*) and LC2/ad (Fig. S1*A*) lung cancer cell lines. We also detected TGF-β-dependent increase of endogenous JUN and JUNB but not of JUND protein in A549 (Fig. 1*B*) and LC2/ad (Fig. S1*B*) cells by immunoblotting. These results suggested the possible involvement of JUN and JUNB TFs in TGF-β-induced EMT process.

Next, we examined the knockdown effects of JUN family members in A549 and LC2/ad cells to clarify their functions in EMT process. We used two different shRNAs (shRNA#1 and #2) for each gene and confirmed that the two shRNAs similarly decreased the expression of the target gene in both cells by QRT–PCR (Fig. S2). During EMT, epithelial cells acquire a mesenchymal phenotype by downregulating an epithelial cell marker, *CDH1*, and upregulating mesenchymal cell markers such as *VIM*, *FN1*, and *CDH2* (3). We analyzed the expression levels of them by QRT–PCR (Figs. 1, *C*–*E*, S3, and S4). The two shRNAs for each gene caused similar effects in the expression of EMT-related genes in both cells (Figs. S3 and S4), and therefore, the data of shRNA#1 in A549 cells were shown as the representative results in the main figures (Fig. 1, *C*–*E*). Knockdown of *JUN* and *JUNB* significantly increased the expression of an epithelial marker gene, *CDH1*, in the absence of TGF-β, and partly inhibited its transcriptional repression mediated by TGF-β in A549 and LC2/ad cells (Figs. 1, *C*, *D*, S4, *A* and *B*). This result strongly suggested the important contribution of JUN and JUNB TFs to the transcriptional regulation during EMT of lung cancer cells. Among the mesenchymal marker genes, *JUN* and *JUNB* knockdown had no influence on the expression of *VIM* gene without TGF-β but slightly prevented TGF-β-dependent increase of its expression in both cells (Figs. 1, *C*, *D*, S4, *A* and *B*). For *FN1* gene, knockdown of *JUNB* but not of *JUN* significantly reduced its expression level in the presence or the absence of TGF-β in A549 cells (Fig. 1, *C* and *D*), although *FN1* expression was too low to be analyzed in LC2/ad cells. On the contrary, knockdown of *JUN* but not of *JUNB* decreased the expression of *CDH2* in both cells (Figs. 1, *C*, *D*, S4, *A*, and *B*), indicating the different functions of JUN and JUNB TFs in the regulation of mesenchymal marker genes. In contrast, knockdown of *JUND* did not show any significant effects on the expression of these EMT-related genes (Figs. S1*E* and S4*C*), indicating little contribution of JUND TF to EMT of lung cancer cells.

We also examined these inhibitory effects of *JUN* or *JUNB* knockdown on the protein expression of E-cadherin, vimentin, fibronectin, and N-cadherin during TGF-β-induced EMT of A549 and LC2/ad cells (Figs. 1, *F*, *G*, S5, *A* and *B*). Efficient downregulation of JUN or JUNB protein slightly increased E-cadherin expression and significantly inhibited its TGF-β-mediated reduction. We could confirm that *JUNB* knockdown reduced the TGF-β-dependent increase of fibronectin (Fig. 1*G*), whereas *JUN* knockdown inhibited the N-cadherin induction by TGF-β (Figs. 1*F* and S5*A*). These results indicated that JUN and JUNB TFs controlled the expression of common or specific EMT-related target genes and played a crucial role in TGF-β-induced EMT of A549 and LC2/ad lung cancer cell lines.

### The expression of JUN was regulated by m6A methyltransferase METTL3 at the protein level

**Figure 2:**
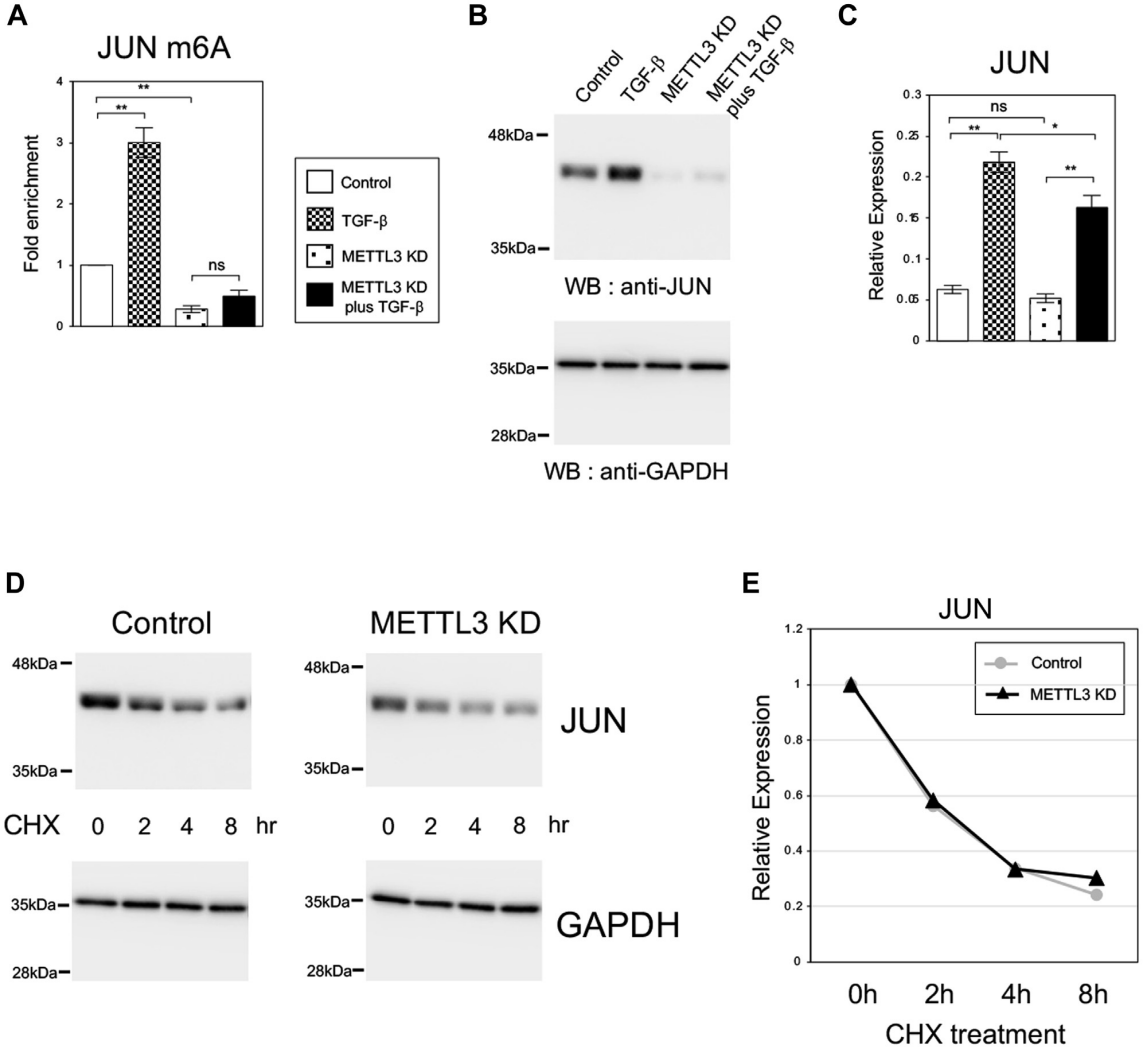
**Knockdown of *METTL3* decreased the m6A modification of *JUN* mRNA and the expression of JUN protein but did not affect the stability of JUN protein in A549 cells.***A*, the m6A-methylated *JUN* mRNA level in the *METTL3* knockdown A549 cells. The m6A-IP–QPCR assay was performed in the control or *METTL3* knockdown A549 cells with or without TGF-β for 24 h (n = 3) (∗∗*p* < 0.01; ns, not significant). *B*, the JUN protein level in the *METTL3* knockdown cells. Immunoblotting was performed to detect JUN protein in the cells shown in (*A*). As a control, anti-GAPDH antibody was used. *C*, the total *JUN* mRNA level in the *METTL3* knockdown cells. QRT–PCR for *JUN* was performed in the cells shown in (*A*) (n = 3) (∗∗*p* < 0.01; ∗*p* < 0.05; ns, not significant). *D* and *E*, the protein stability of JUN in the *METTL3* knockdown cells. The control and *METTL3* knockdown A549 cells were treated with cycloheximide (CHX) for the indicated times, and protein expression of JUN was analyzed by immunoblotting (*D*). The band intensities were measured, and the quantitative values of JUN protein normalized by GAPDH expression were plotted (*E*). IP, immunoprecipitation; m6A, N6-methyladenosine; METTL, methyltransferase-like; QPCR, quantitative PCR.

Previously, we have demonstrated that JUNB is one of the important TFs regulated by METTL3 m6A methyltransferase during TGF-β-induced EMT process (21). This finding triggered us to investigate whether JUN was controlled by METTL3 in A549 and LC2/ad cells. We examined the m6A methylation level of *JUN* mRNA by m6A RNA immunoprecipitation (m6A RIP) assay. The m6A-modified *JUN* mRNA was increased by TGF-β treatment in A549 and LC2/ad lung cancer cells (Figs. 2*A* and S6*A*). *METTL3* knockdown decreased the m6A methylation of *JUN* mRNA in the absence of TGF-β and cancelled the TGF-β effect on the increase of m6A level (Fig. 2*A* and S6*A*). Since METTL3 has been shown indispensable for TGF-β-induced EMT (21), m6A methylation of *JUN* mRNA was suggested to play an important regulatory role in EMT. We also found that *METTL3* knockdown reduced the protein level of JUN in both cells and cancelled the TGF-β-mediated induction of JUN protein (Figs. 2*B* and S6*B*). Interestingly, *METTL3* knockdown had no effect on *JUN* mRNA expression in the absence of TGF-β and decreased TGF-β-dependent induction only slightly (Figs. 2*C* and S6*C*). Therefore, the reduction of JUN protein by *METTL3* knockdown could not be explained by the mRNA expression level. These results indicated that METTL3-mediated m6A methylation influenced the expression of JUN protein but not of *JUN* mRNA. This is in contrast with the case of m6A-dependent regulation of JUNB, since a decrease in *JUNB* mRNA was clearly detected by *METTL3* knockdown (21). Then, we hypothesized that the reduction of JUN protein in the *METTL3* knockdown cells might be due to the difference in protein stability or translational regulation. To examine the stability of JUN protein, the control and *METTL3* knockdown cells were treated with cycloheximide to block protein synthesis. Then immunoblotting was performed at the indicated period after treatment in A549 and LC2/ad cells (Figs. 2*D* and S6*D*). Quantitative analysis of the band intensities revealed that the expression of JUN protein was similarly decreased in the control and *METTL3* knockdown cells (Figs. 2*E* and S6*E*), indicating that protein stability of JUN was not influenced by METTL3-dependent m6A regulation. These results together suggested that the m6A-mediated expression of JUN protein was related to the regulation of translation process rather than the mRNA regulation and protein stability.

### JUNB mRNA was regulated by the m6A modifications at its 3′UTR

**Figure 3:**
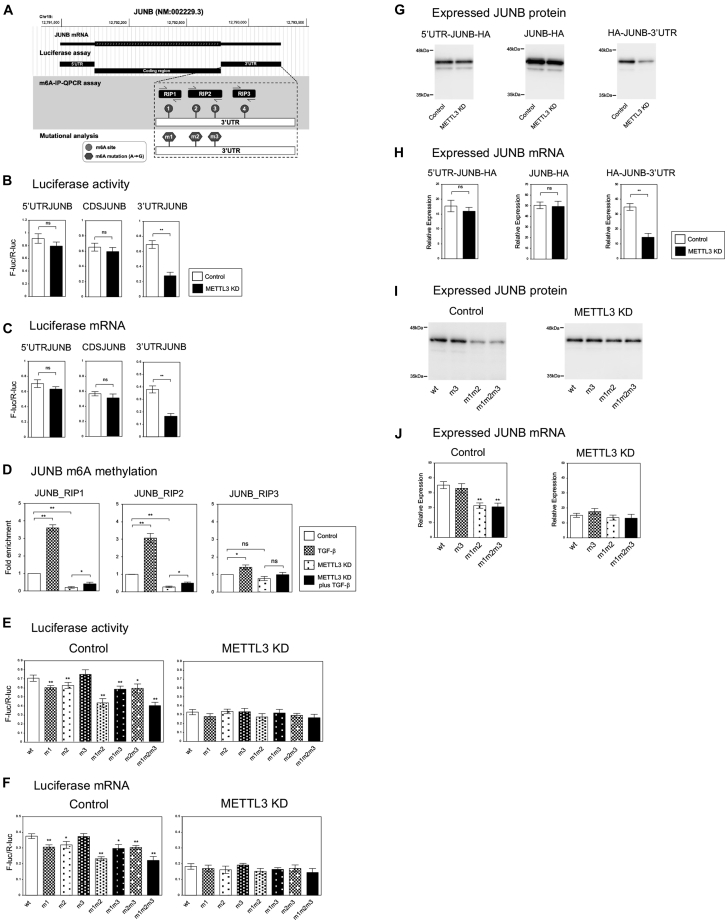
**METTL3 regulated *JUNB* mRNA through the m6A methylation sites within *JUNB* 3′ UTR.***A*, schematic representation of the structure, cloned fragments, PCR amplicons, m6A motifs, and mutants of *JUNB* cDNA. *B* and *C*, luciferase assays to determine the region of *JUNB* responsible for its downregulation by *METTL3* knockdown. The pmirGLO plasmids containing 5′UTR, coding region sequence (CDS), and 3′UTR of *JUNB* were transfected into the control and *METTL3* knockdown A549 cells. The Firefly and Renilla luciferase activities were measured, and the relative activities of F-luc/R-luc were shown (*B*) (n = 4) (∗∗*p* < 0.01; ns, not significant). QRT–PCR was performed to detect the mRNAs of Firefly and Renilla luciferase, and the relative units of F-luc/R-luc were similarly presented (*C*). *D*, the m6A methylated levels of *JUNB* 3′UTR regions. The m6A-IP–QPCR assay using fragmented RNA was performed in the control or *METTL3* knockdown A549 cells with or without TGF-β. Fold enrichment was shown for the indicated PCR amplicons located in 3′UTR of *JUNB* (n = 3) (∗∗*p* < 0.01; ∗*p* < 0.05; ns, not significant). *E* and *F*, luciferase assays for mutational analysis of *JUNB* m6A sites related to m6A regulation. The pmirGLO plasmids containing the indicated *JUNB* 3′UTR m6A mutations were transfected into the control and *METTL3* knockdown A549 cells. The relative luciferase activities (*E*) and mRNA levels (*F*) of F-luc/R-luc were presented (n = 4) (∗∗*p* < 0.01; ∗*p* < 0.05; ns, not significant). *G* and *H*, exogenous JUNB expression assays to confirm the responsible region. HA-tagged JUNB expression plasmid containing 5′UTR, 3′UTR, or no UTR was transfected into the control and *METTL3* knockdown A549 cells with R-luc expression plasmid. Immunoblotting (*G*) and QRT–PCR (*H*) to detect exogenous HA-tagged JUNB protein and mRNA were shown (n = 3) (∗∗*p* < 0.01; ns, not significant). Transfection efficiencies were normalized by the R-luc expressions and activities. *I* and *J*, exogenous JUNB expression assays to confirm the mutational analysis of m6A sites. HA-tagged JUNB expression plasmids containing the indicated 3′UTR m6A mutations were transfected into the cells. Immunoblotting (*I*) and QRT–PCR (*J*) to detect HA-JUNB protein and mRNA were shown (n = 3) (∗∗*p* < 0.01; ns, not significant). cDNA, complementary DNA; HA, hemagglutinin; IP, immunoprecipitation; m6A, N6-methyladenosine; METTL, methyltransferase-like; QRT–PCR, quantitative RT–PCR.

In the previous study, we reported that the stability of *JUNB* mRNA was regulated through m6A methylation by METTL3 during EMT process (21). To further investigate the mechanism, we tried to determine which region of *JUNB* was responsible for the m6A-dependent regulation. We constructed dual luciferase reporter plasmids containing a Firefly luciferase followed by 5′UTR, coding sequence (CDS), or 3′UTR of *JUNB* (Fig. 3*A*) and an internal control Renilla luciferase. These plasmids were transfected into the control and *METTL3* knockdown A549 cells, and luciferase activity and the expression of luciferase mRNA were analyzed. Among the three types of reporter plasmids, the plasmid containing *JUNB* 3′UTR only showed reduced luciferase activity in the *METTL3* knockdown cells than in the control cells (Fig. 3*B*). QRT–PCR revealed a similar decrease of luciferase mRNA only for the reporter with *JUNB* 3′UTR (Fig. 3*C*). These results indicated that 3′UTR of *JUNB* contained the responsible region for mRNA regulation by METTL3 enzyme.

According to the database of m6A methylation sites (ConsRM) (22), there are four potential m6A methylation motifs (m6A_1–4) within 3′UTR of *JUNB* complementary DNA (cDNA) in A549 cells (Fig. 3*A*). We designed the three sets of PCR primers to amplify the fragments containing these m6A sites and performed m6A RNA-IP for fragmented RNA isolated from the control and *METTL3* knockdown A549 cells with or without TGF-β (Fig. 3*D*). QRT–PCR revealed that all amplified regions (JUNB_RIP1, 2, and 3) showed a significant increase of m6A level in the presence of TGF-β. The RIP1 and RIP2 but not RIP3 amplicons detected a reduction of m6A level in the *METTL3* knockdown cells (Fig. 3*D*). This result strongly suggested that the RIP1 and RIP2 regions of *JUNB* 3′UTR contained the m6A sites controlled by METTL3. Since there are three potential m6A sites (m6A_1–3) within the RIP1 and RIP2 regions, we generated the luciferase reporter plasmids for each m6A site mutant (m1, m2, and m3) and the combined mutants (Fig. 3*A*). These plasmids were transfected into the control and *METTL3* knockdown A549 cells, and luciferase activity and mRNA expression were analyzed. The m1 and m2 mutants of m6A sites resulted in a significant decrease of luciferase activity and mRNA expression in the control cells, and the combined mutant (m1m2) showed further reduction (Fig. 3, *E* and *F*). However, the m3 mutant showed no difference in luciferase activity and mRNA, and the m3 mutation did not cause any further effects even when it was combined with others (Fig. 3, *E* and *F*). Notably, the decrease of luciferase activity and mRNA by the m1 and m2 mutants was not detected in the *METTL3* knockdown cells (Fig. 3, *E* and *F*), indicating the requirement of METTL3 enzyme in the regulation through the m6A sites. These results suggested that the m6A_1 and m6A_2 sites of *JUNB* 3′UTR were the target sites of METTL3 for the regulation of *JUNB* mRNA.

Next, we confirmed the results obtained from the luciferase reporter constructs in a different way. We constructed hemagglutinin (HA)-tagged JUNB expression plasmids containing 5′UTR-CDS, only CDS, or CDS-3′UTR (Fig. 3*A*) and transfected them into the control and *METTL3* knockdown A549 cells. The Renilla luciferase plasmid was cotransfected to normalize the transfection efficiency. Immunoblotting and QRT–PCR analysis revealed that the construct containing JUNB CDS-3′UTR only showed a remarkable decrease of JUNB protein (Fig. 3*G*) and mRNA (Fig. 3*H*) in the *METTL3* knockdown cells, indicating the importance of 3′UTR in METTL3-mediated regulation. Then we used JUNB CDS-3′UTR expression plasmids for wildtype and the selected m6A site mutants (m3, m1m2, and m1m2m3) in the similar experiment. We confirmed that the m1 and m2 mutations caused a reduction of JUNB protein and mRNA, but the m3 mutation had no effect in the control cells (Fig. 3, *I* and *J*). Again, this m6A site–dependent regulation of JUNB expression was not observed in the *METTL3* knockdown cells (Fig. 3, *I* and *J*). Taken together, we concluded that the m6A_1 and m6A_2 sites of *JUNB* 3′UTR were responsible for m6A-mediated regulation of *JUNB* mRNA in A549 lung cancer cells.

### The expression of JUN protein was regulated by the m6A modifications of its mRNA at 3′UTR

**Figure 4:**
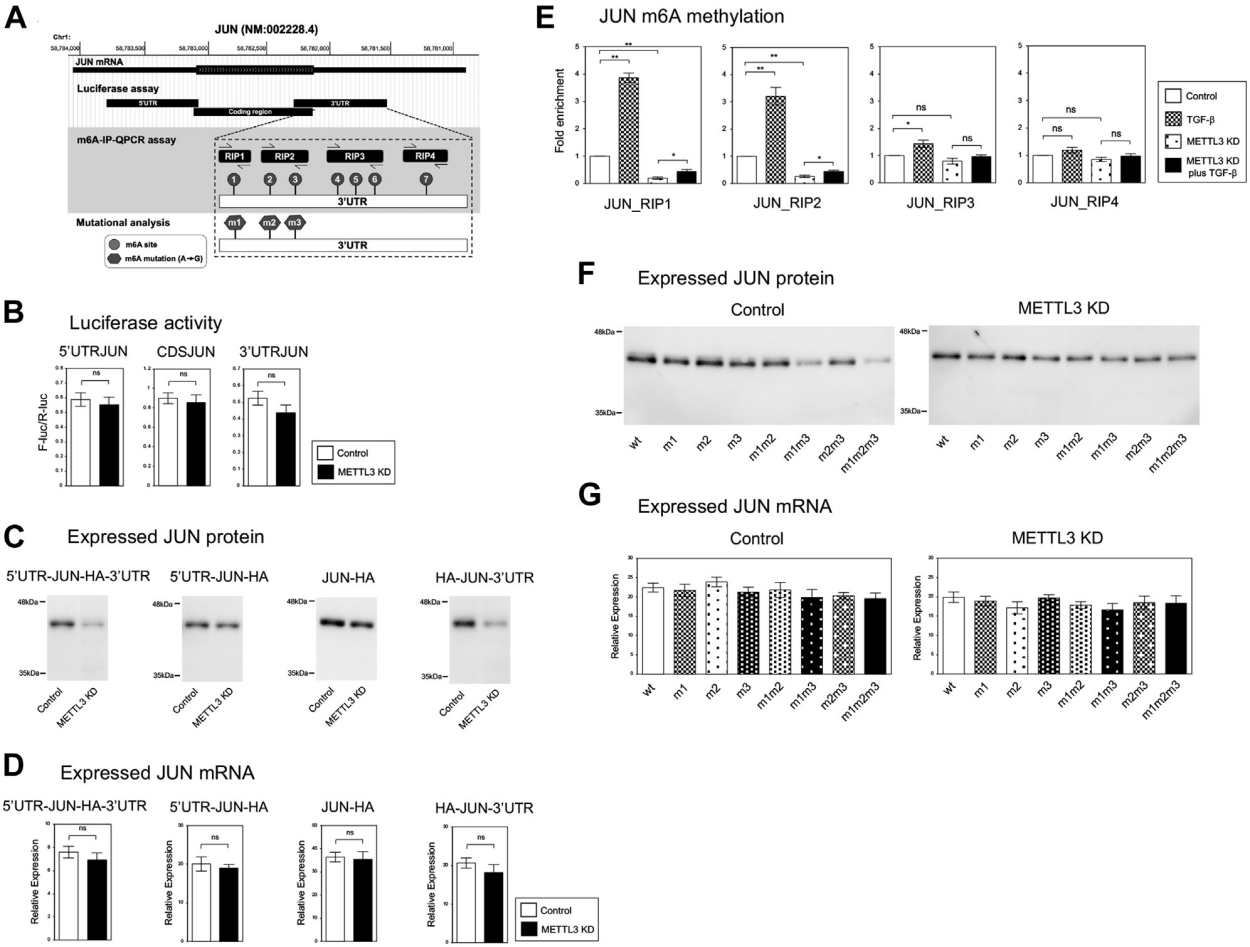
**METTL3 regulated JUN protein through the m6A methylation sites within *JUN* 3′ UTR.***A*, schematic representation of the structure, cloned fragments, PCR amplicons, m6A motifs, and mutants of *JUN* cDNA. *B*, Luciferase assays to determine the region of *JUN* responsible for the downregulation by *METTL3* knockdown. The pmirGLO plasmids containing 5′UTR, coding region, and 3′UTR of *JUN* were transfected into the control and *METTL3* knockdown A549 cells. The relative luciferase activities of F-luc/R-luc were shown (n = 4) (ns, not significant). *C* and *D*, exogenous JUN expression assays to determine the responsible region. HA-tagged JUN expression plasmid containing 5′UTR, 3′UTR, both, or no UTR was transfected into the control and *METTL3* knockdown A549 cells with R-luc expression plasmid. Immunoblotting (*C*) and QRT–PCR (*D*) to detect exogenous HA-tagged JUN protein and mRNA were shown (n = 3) (ns, not significant). Transfection efficiencies were normalized by the R-luc expressions and activities. *E*, the m6A methylated levels of *JUN* 3′UTR regions. The m6A-IP–QPCR assay using fragmented RNA was performed in the control or *METTL3* knockdown A549 cells with or without TGF-β. Fold enrichment was shown for the indicated PCR amplicons located in 3′UTR of *JUN* (n = 3) (∗∗*p* < 0.01; ∗*p* < 0.05; ns, not significant). *F* and *G*, exogenous JUN expression assays for the mutational analysis of *JUN* m6A sites. HA-tagged JUN expression plasmids containing the indicated 3′UTR m6A mutations were transfected into the cells. Immunoblotting (*F*) and QRT–PCR (*G*) to detect HA-JUN protein and mRNA were shown (n = 3). cDNA, complementary DNA; m6A, N6-methyladenosine; METTL, methyltransferase-like; HA, hemagglutinin; IP, immunoprecipitation; QPCR, quantitative PCR; TGF-β, transforming growth factor-beta.

We next tried to determine which region of *JUN* mRNA was involved in the regulation of protein expression by METTL3 enzyme. We constructed luciferase reporter plasmids containing 5′UTR, CDS, or 3′UTR of *JUN* (Fig. 4*A*) and examined the luciferase activities in the control and *METTL3* knockdown A549 cells. However, we did not detect any significant differences (Fig. 4*B*). Then we utilized HA-tagged JUN expression plasmids containing 5′UTR-CDS-3′UTR, 5′UTR-CDS, only CDS, or CDS-3′UTR (Fig. 4*A*) to compare the expression levels between the control and *METTL3* knockdown cells. As shown in Figure 4*C*, the plasmids containing 5′UTR-CDS-3′UTR and CDS-3′UTR but not others revealed decreased expression of JUN protein in the *METTL3* knockdown cells. QRT–PCR showed no significant differences of *JUN* mRNA for all the constructs (Fig. 4*D*). This result was consistent with the finding that JUN expression was controlled by METTL3 at the protein level (Fig. 2). These results indicated that 3′UTR of *JUN* contained the region critical for the regulation by METTL3 enzyme.

The database of m6A methylation sites (22) revealed seven potential m6A motifs (m6A_1–7) within 3′UTR of *JUN* cDNA in A549 cells (Fig. 4*A*). We designed the four PCR primer pairs (JUN_RIP1 to 4) to cover these m6A sites and performed m6A RNA-IP for fragmented RNA isolated from the indicated cells (Fig. 4*E*). QRT–PCR indicated that the JUN_RIP1 and RIP2 amplicons but not others showed a remarkable increase of m6A level by TGF-β and a significant decrease of m6A in the *METTL3* knockdown cells (Fig. 4*E*), suggesting that these regions contained the target m6A sites. We found three potential m6A sites (m6A_1–3) within the RIP1 and RIP2 regions and constructed JUN CDS-3′UTR expression plasmids for wildtype, each m6A site mutant (m1, m2, and m3) and the combined mutants (Fig. 4*A*). Immunoblotting showed that combination of m1 and m3 mutations resulted in an obvious reduction of JUN protein in the control cells (Fig. 4*F*). The effect of either m1 mutation or m3 mutation seemed marginal, but the m2 mutation did not cause any effects by itself or combined (Fig. 4*F*). We confirmed that these m6A mutations did not induce any significant differences in the expression of JUN mRNA (Fig. 4*G*). The m6A site–dependent regulation of JUN protein was not observed in the *METTL3* knockdown cells (Fig. 4, *F* and *G*), indicating the requirement of METTL3 in the regulation through the m6A sites. Taken together, we concluded that the m6A_1 and m6A_3 sites of *JUN* 3′UTR were responsible for METTL3-mediated regulation of JUN protein expression in A549 lung cancer cells.

### Different m6A reader proteins were involved in the m6A-dependent regulation of JUN and JUNB

**Figure 5:**
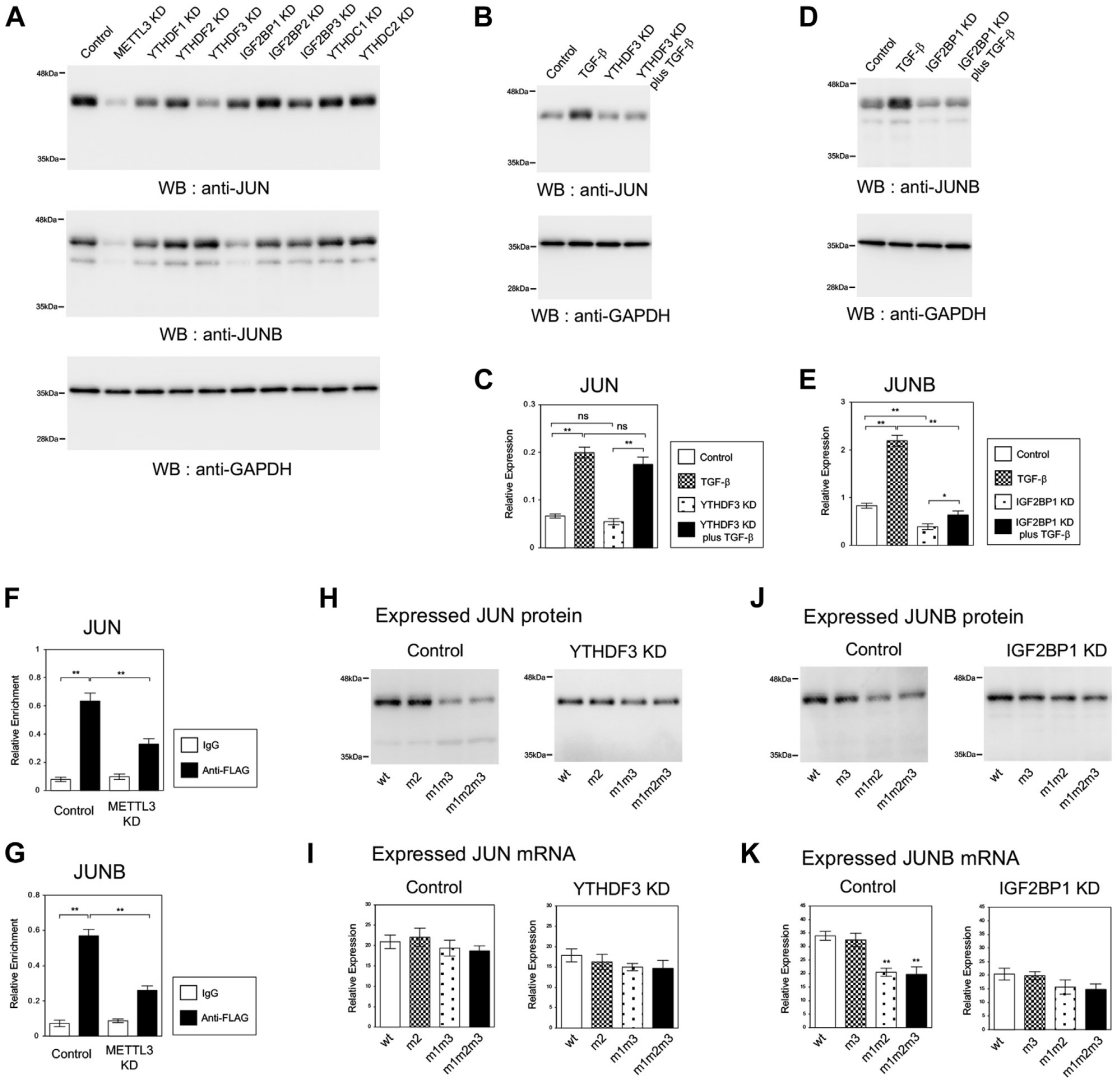
**The m6A reader proteins, YTHDF3 and IGF2BP1, were involved in the m6A-dependent regulation of *JUN* and *JUNB* expression, respectively.***A*, the expression of JUN and JUNB protein in the cells with the knockdown of various m6A readers. Immunoblotting of JUN and JUNB was performed in the cells with the knockdown of *METTL3*, *YTHDF1*, *2*, *3*, *IGF2BP1, 2*, *3*, and *YTHDC1* and *2* in the presence of TGF-β. *B* and *C*, the expression of JUN protein (*B*) and *JUN* mRNA (*C*) in the *YTHDF3* knockdown cells. Immunoblotting was performed to detect JUN protein in the control or *YTHDF3* knockdown A549 cells with or without TGF-β (*B*). As a control, anti-GAPDH antibody was used. QRT–PCR for *JUN* was performed in the same set of cells (*C*) (n = 3) (∗∗*p* < 0.01; ∗*p* < 0.05; ns, not significant). *D* and *E*, the expression of JUNB protein (*D*) and *JUNB* mRNA (*E*) in the *IGF2BP1* knockdown A549 cells. *F*, YTHDF3 RIP–QRT–PCR analysis of *JUN* mRNA. The crosslinked cell lysates were prepared from the control or *METTL3* knockdown A549 cells expressing FLAG-YTHDF3. RIP by normal IgG or anti-FLAG antibody was performed, and the precipitated RNA was analyzed by QRT–PCR with *JUN*_RIP1 primers (n = 3) (∗∗*p* < 0.01). *G*, IGF2BP1 RIP–QRT–PCR analysis of *JUNB* mRNA. The control or *METTL3* knockdown A549 cells expressing FLAG-IGF2BP1 were analyzed by RIP with normal IgG or anti-FLAG antibody, and QRT–PCR was performed with *JUNB*_RIP1 primers (n = 3) (∗∗*p* < 0.01). *H* and *I*, the expression of m6A-site mutants of *JUN* in the *YTHDF3* knockdown cells. HA-tagged JUN expression plasmids containing the indicated 3′UTR m6A mutations were transfected into in the control or *YTHDF3* knockdown A549 cells. Immunoblotting (*H*) and QRT–PCR (*I*) to detect HA-JUN protein and mRNA were shown (n = 3). *J* and *K*, the expression of m6A-site mutants of *JUNB* in the *IGF2BP1* knockdown cells. Immunoblotting (*J*) and QRT–PCR (*K*) to detect HA-JUNB protein and mRNA were presented (n = 3) (∗∗*p* < 0.01; ns, not significant). HA, hemagglutinin; IgG, immunoglobulin G; m6A, N6-methyladenosine; QRT–PCR, quantitative RT–PCR; RIP, RNA immunoprecipitation; TGF-β, transforming growth factor-beta.

Our results indicated that METTL3 controlled the expression of *JUNB* at the mRNA level but controlled *JUN* expression at the protein level. We hypothesized that different m6A reader proteins might be involved in the m6A-mediated regulation of *JUN* and *JUNB*. To find the candidate m6A reader proteins for *JUN* and *JUNB*, we examined the effect of shRNA-mediated knockdown of each m6A reader on the expression of JUN and JUNB proteins in A549 lung cancer cells (Fig. 5*A*). Efficient knockdown of eight representative m6A readers (YTHDF1, YTHDF2, YTHDF3, IGF2BP1, IGF2BP2, IGF2BP3, YTHDC1, and YTHDC2) by the corresponding shRNA was confirmed by QRT–PCR and immunoblotting (Fig. S7, *A* and *B*). We performed immunoblotting to detect JUN and JUNB proteins in each m6A reader knockdown cells and compared the band intensities with those in the control (negative control) and *METTL3* knockdown (positive control) cells. The results showed that JUN protein was remarkably downregulated in *YTHDF3* knockdown cells, and JUNB protein was decreased with *IGF2BP1* knockdown (Fig. 5*A*). We decided to pick up YTHDF3 and IGF2BP1 as the candidate m6A reader proteins for *JUN* and *JUNB*, respectively.

To validate the involvement of YTHDF3 and IGF2BP1 in the regulation of JUN and JUNB expression, we confirmed the knockdown effects in protein and mRNA expression in A549 and LC2/ad lung cancer cells with or without TGF-β treatment. We found that *YTHDF3* knockdown reduced the protein level of JUN in A549 (Fig. 5*B*) and LC2/ad cells (Fig. S8*A*) and almost canceled the TGF-β-dependent induction of JUN protein. Interestingly, *YTHDF3* knockdown had no effect on JUN mRNA expression in the presence or the absence of TGF-β (Figs. 5*C* and S8*B*). In contrast, *IGF2BP1* knockdown decreased the expression and induction of JUNB protein and mRNA in A549 (Fig. 5, *D* and *E*) and LC2/ad (Fig. S8, *C* and *D*) cells. These results are consistent with the aforementioned results for the m6A-dependent regulation of JUN protein and *JUNB* mRNA.

To detect the interaction between the m6A reader proteins and *JUN* or *JUNB* mRNA, we performed RIP–QRT–PCR analysis. The control or *METTL3* knockdown A549 cells were infected with retroviruses expressing FLAG-tagged YTHDF3, and the crosslinked cell lysates were prepared in the presence of TGF-β. RIP was performed with normal immunoglobulin G (IgG) or anti-FLAG antibody, and the coprecipitated RNA was analyzed by QRT–PCR with JUN_RIP1 primers (Fig. 5*F*). The interaction of FLAG-YTHDF3 protein and *JUN* mRNA was clearly detected in the control cells with YTHDF3 overexpression, but this enrichment was significantly reduced in the *METTL3* knockdown cells (Fig. 5*F*). Similar RIP–QRT–PCR experiments were carried out using FLAG-IGF2BP1 overexpression and JUNB_RIP1 primers (Fig. 5*G*). We observed the interaction between FLAG-IGF2BP1 and *JUNB* mRNA, which was decreased by *METTL3* knockdown (Fig. 5*G*). These results indicated the significant interactions of YTHDF3 protein/JUN mRNA and IGF2BP1 protein/JUNB mRNA in lung cancer cells and suggested that these interactions were dependent on the m6A modifications by METTL3.

Next, we transfected JUN CDS-3′UTR expression plasmids for wildtype and the selected m6A site mutants (m2, m1m3, and m1m2m3) into the *YTHDF3* knockdown cells. We could not observe the m1/m3 mutation–dependent reduction of JUN protein in the *YTHDF3* knockdown cells (Fig. 5, *H* and *I*). Similarly, JUNB mutant analysis revealed that the m6A site–dependent regulation of *JUNB* mRNA was not detected in the *IGF2BP1* knockdown cells (Fig. 5, *J* and *K*). These results indicated the requirement of YTHDF3 and IGF2BP1 in the m6A-mediated regulation of JUN and JUNB, respectively. Therefore, our candidate approach revealed that YTHDF3 and IGF2BP1 were the most reasonable candidate m6A reader proteins involved in the regulation of expression of JUN protein and *JUNB* mRNA, respectively.

## Discussion

In this study, we discovered the m6A RNA modification–dependent regulation of JUN and JUNB TFs, which plays an essential and nonredundant role in gene expression program during TGF-β-induced EMT of A549 and LC2/ad lung cancer cells. Mechanistic investigations strongly suggested that METTL3 enzyme regulated the translation process of JUN protein and the stability of *JUNB* mRNA through the m6A motifs located at 3′UTR of *JUN* and *JUNB*. In addition, YTHDF3 and IGF2BP1 were identified as the most reasonable candidate m6A reader proteins for JUN and JUNB, respectively. We concluded that the different modes of m6A-mediated regulation of JUN and JUNB TFs during EMT process could be attributed to the distinct functions of these m6A reader proteins.

JUN family TFs control the expression of downstream target genes and take part in the regulation of cellular proliferation, apoptosis, and malignant transformation (16). Previous studies indicate that each member of JUN family contributes to EMT process in different types of cancer cells, thereby affecting cell migration and invasion activities (17, 18, 19, 20). Since the target genes controlled by each JUN family member and the modes of regulation are different, the underlying molecular mechanisms operating during EMT are predicted to be different. In this study, we found that knockdown of *JUN* and *JUNB* affected the expression of epithelial and mesenchymal marker genes during TGF-β-dependent EMT of A549 and LC2/ad lung cancer cells (Figs. 1, S3, S4 and S5). However, *JUND* knockdown had no effects on the EMT-related genes as far as we examined, suggesting the functional importance of the induced expression of *JUN* and *JUNB* by TGF-β (Figs. 1, *A*, *B* and S1). More importantly, our data revealed that JUN and JUNB TFs controlled the expression of different mesenchymal marker genes, *CDH2* and *FN1*, respectively (Figs. 1, S3, S4 and S5). This is well correlated with the distinct gene regulatory activities of JUN and JUNB proposed by the previous reports (17, 19). Overall, our results demonstrated that both common and individual functions of JUN and JUNB TFs were important in the transcriptional regulation during EMT of lung cancer cells. This is also consistent with the previous studies suggesting that EMT-related TFs have overlapping and nonoverlapping functions in EMT process, which can be specific to the cancer types (4). However, we now remain far from understanding the complex gene regulatory network in EMT controlled by the different EMT-related TFs. To address their roles in EMT, a comprehensive analysis of the transcriptome and epigenome may be required by incorporating new technologies such as chromatin immunoprecipitation sequencing, assay for transposase-accessible chromatin using sequencing, and single-cell RNA-Seq.

Accumulating evidence in recent years revealed that m6A modification by METTL3 enzyme participates in many pathological processes including cancer (13, 14, 15). Other groups reported the important function of METTL3 and its m6A-modified targets in various cancer types. For example, METTL3 regulates *SOX2* mRNA in glioma stem cell maintenance and colorectal tumor progression (23, 24), *LEF1* in osteosarcoma progression (25), *AFF4/NF-kB/MYC* in bladder cancer (26), and *SPHK2* in gastric cancer (27). Especially, with regard to EMT process, several articles demonstrated the important roles of m6A regulation on *SNAI1* in HeLa and HepG2 cells (28), *GFI1* and *ZMYM1* in gastric cancer cells (29, 30), *AXL* in ovarian cancer cells (31), and *ZBTB1* in bronchial epithelial cells (32). We have so far demonstrated that the m6A regulation of *JUN* and *JUNB* by METTL3 plays a critical role in the expression levels of them, thereby affecting the EMT-inducing gene expression program of A549 and LC2/ad lung cancer cells. However, the observed phenotypic inhibition of EMT by *METTL3* knockdown (21) cannot be fully explained by the reduced expression of JUN and JUNB TFs. Further examinations for other EMT-related TFs and cellular factors controlled by METTL3 are warranted to elucidate the precise mechanism for the METTL3 function in EMT of lung cancer. In addition, the levels of RNA m6A modification are controlled by m6A methyltransferases and demethylases. Thus, we examined the expression levels of ALKBH5 and FTO m6A demethylases in TGF-β-induced EMT of A549 and LC2/ad cells (Fig. S9). QRT–PCR and immunoblotting were performed similarly in the case of JUN family (Figs. 1, *A*, *B*, S1, *A* and *B*). The expression levels of ALKBH and FTO mRNAs and proteins were not changed in response to TGF-β in both cells (Fig. S9). We observed a slight decrease in their expressions after TGF-β treatment, but the differences were not statistically significant (Fig. S9). In the previous study (21), we detected a significant increase in the expression of METTL3 during EMT of A549 and LC2/ad cells. These results suggest that METTL3 m6A methyltransferase may contribute mainly to the increased m6A modification of mRNAs in TGF-β-induced EMT of lung cancer cells.

To determine the responsible regions of *JUNB* and *JUN* for the m6A regulation by METTL3, we used luciferase reporter plasmids and HA-tagged cDNA expression plasmids containing different parts of each cDNA. We could find *JUNB* 3′UTR as the m6A regulatory region in both methods (Fig. 3) and *JUN* 3′UTR as the responsible region only in the HA-tagged cDNA expression system (Fig. 4). We do not know the exact reason for the failure in the luciferase reporter of *JUN*. Still, we hypothesize that the original CDS of *JUN* may be required for the protein regulation by m6A modification. Eventually, we have identified the two specific m6A sites for *JUN* and *JUNB*, respectively, which are closely associated with the m6A-dependent regulation of JUN protein and *JUNB* mRNA (Figs. 3 and 4). Since these results are derived from the experiments based on the candidate m6A motifs registered in the database (22), we cannot exclude the possibility that other unexamined m6A sites might be involved. However, the mutation analysis for each or combined m6A sites strongly supported the functional importance of these m6A sites in the regulation of JUN and JUNB.

In this study, we focused on YTHDF3 and IGF2BP1 as the candidate m6A reader proteins for JUN and JUNB, respectively, because the knockdown experiment revealed the most remarkable effect on the expression (Fig. 5). Since only eight m6A reader proteins have been examined in our candidate approach, we cannot rule out the possibility that other m6A reader proteins may be involved. However, our RIP–QRT–PCR assay and m6A mutant analysis convinced us to conclude that YTHDF3 and IGF2BP1 were the most reasonable candidate m6A reader proteins for JUN and JUNB, respectively (Fig. 5). YTHDF3 belongs to YTH domain–containing protein family (YTHDF1–3, YTHDC1, and YTHDC2), which has been recognized as “Readers” to specifically identify the m6A-modified mRNAs and control their stability, splicing, exportation, and translation (33). Several studies on YTHDF3 have revealed its function to promote translation efficiency dependent on m6A modification (33). These reports strongly support our result that YTHDF3 is the m6A reader for JUN, since m6A-dependent expression of JUN protein appears to be associated with the regulation of translation process (Figs. 2 and S6). YTHDF3 shares some targets with YTHDF1 and functions through interacting with YTHDF1 (34). We also observed slight reduction of JUN protein expression in the *YTHDF1* knockdown cells (Fig. 5*A*), which might reflect the additional contribution of YTHDF1 to the m6A regulation of JUN. As the potential mechanism of enhanced translation, it has been reported that YTHDF3–YTHDF1 interact with the 40S and 60S ribosomal subunits (35) and the translation initiation factor, eIF4A3 (34). Therefore, it would be the next important subject to elucidate the mechanism of YTHDF3-mediated regulation of JUN protein expression during EMT. Recently, YTHDF3 has been shown to be associated with the progression of several types of tumors. YTHDF3 promoted translation of CTNNB1, contributing to proliferation, migration, and maintenance of cancer stem–like properties in ocular melanoma (36), and mediated breast cancer brain metastasis through increasing m6A-dependent ST6GALNAC5 and epidermal growth factor receptor expressions (37). Interestingly, in triple-negative breast cancer cells, YTHDF3 was involved in EMT, cell migration, and invasion (38). The expression of E-cadherin was increased, whereas N-cadherin and vimentin expressions were decreased through the ZEB1 mRNA destabilization by YTHDF3 knockdown (38). These studies suggest that the function of YTHDF3 and the target mRNAs it controls have not been fully explored yet. Thus, it is important to accumulate the evidence indicating the common or diverse function of YTHDF3 in the malignant progression of cancer including EMT.

IGF2BP1 is a member of IGF2BP family (IGF2BP1–3), which recognizes m6A and facilitates m6A-modified mRNA stabilization. This is consistent with our results indicating that IGF2BP1 controls the m6A-dependent expression of *JUNB* mRNA. IGF2BP1 is originally reported as an oncofetal protein, which is expressed in embryonic tissues and various types of tumors but is downregulated in normal adult tissues (39). The functions of IGF2BP1 in oncogenesis have been extensively studied even before it was recognized as an m6A reader protein (39). Recently, it has been reported that IGF2BP1 promoted cell growth, migration, and invasion of hepatocellular carcinoma cells and cervical cancer cells by stabilizing the target mRNAs including MYC (40) and controlled stem cell maintenance of ovarian cancer cells by impairing the miRNA-directed decay of serum response factor mRNA (41). However, to date, there have been few studies specifically describing m6A regulation by IGF2BP1 in EMT process. A previous article showed that IGF2BP1 promoted EMT by preventing LEF1 mRNA degradation in human embryonic kidney 293 and U2OS cells, but the m6A dependency remained unknown (42). LEF1 is known to be involved in the transcription of a mesenchymal marker fibronectin (42), which is similar to the case of JUNB in this study. Therefore, it would be interesting to clarify the role of IGF2BP1 in EMT by focusing on its function in the transcriptional regulation of a subset of mesenchymal genes including fibronectin.

In summary, we demonstrated the different modes of m6A regulation for JUN and JUNB TFs, which play an integral role in the EMT-inducing gene regulation during TGF-β-dependent EMT of lung cancer cell lines. Different m6A reader proteins, YTHDF3 and IGF2BP1, were shown to associate with *JUN* and *JUNB* mRNA, respectively, and to mediate the m6A-dependent regulation of JUN protein translation and *JUNB* mRNA stability. Phenotypic plasticity of cancer cells driven by EMT contributes to malignant progression through metastasis and drug resistance. Targeting EMT process has the potential to enhance cancer therapy, and the identification of new molecular targets in EMT is very useful for future clinical applications. We propose that m6A-dependent regulation of EMT-related TFs offers a good therapeutic target in preventing metastasis and overcoming therapy resistance.

## Experimental procedures

### Plasmids, cell culture, and transfections

For the knockdown experiments, lentiviral vectors expressing shRNAs were constructed as described previously (43). The oligonucleotides were synthesized, annealed, and cloned into pLKO.1-Puro plasmid (Sigma–Aldrich). The oligonucleotide sequences for shRNAs were described previously (21) and are listed in Table S1. We usually used two different shRNAs (shRNA#1 and shRNA#2) for each gene. Two shRNAs that have similar efficiencies for knockdown of each gene were selected and used (Figs. S2 and S7). For JUN family, the two shRNAs have similar effects on EMT phenotypes judged from the marker gene expression (Figs. S3 and S4). Thus, the data of one representative shRNA for each gene were shown in the main figures and text.

For the cloning of human *JUN* and *JUNB* cDNA, the primer sets described in Table S1 were designed based on the reference sequences (*JUN*: NM_002228.4 and *JUNB*: NM_002229.3) in National Center for Biotechnology Information database. The amplified cDNAs were cloned into pEF6/HA-His plasmid (a gift from Dr T. Nakamura, Kansai Medical University) or pCG-HA plasmid (44) to express HA-tagged protein. To clone *YTHDF3* and *IGF2BP1* cDNA, we designed the primers (Table S1) based on the reference sequences (*YTHDF3*: NM_152758.6 and *IGF2BP1*: NM_006546.4). The amplified cDNAs were tagged with FLAG as described (44) and then cloned into pDON-5 Neo plasmid (Takara) to produce the retroviruses.

To generate the 5′UTR, CDS, and 3′UTR of *JUN* or *JUNB* luciferase reporter constructs, the DNA fragments were amplified by PrimeSTAR MAX DNA polymerase (catalog no.: R045A; Takara) using each primer set (Table S1) and cloned into NheI and XhoI sites of pmirGLO vector (catalg no.: E1330; Promega).

To construct the expression vectors for m6A site mutants, we used PrimeSTAR Mutagenesis Basal kit (catalog no.: R046A; Takara) according to the manufacturer’s protocol. Each single-point mutation for the three candidate m6A sites was introduced into 3′UTR of *JUN* cDNA using *JUN* m6A mutant primer sets described in Table S1. The combined mutations were created by introducing the different mutations sequentially. The *JUNB* m6A mutations were also introduced by the same procedure using *JUNB* m6A mutant primer sets (Table S1). The nucleotide exchanges of the mutants were confirmed by DNA sequencing. The sequence-verified PCR products were used for cloning and plasmid construction.

Human lung cancer cell lines, A549 and LC2/ad, were purchased from American Type Culture Collection and RIKEN BioResource Research Center and cultured in Dulbecco’s modified Eagle’s medium and the mixture of RPMI1640 and HAMS F12 medium with 10% fetal bovine serum, respectively (8). These two cell lines are a good model system for EMT study showing rapid and clear changes in cell morphology and EMT-related gene expression induced by treatment of 1 ng/ml of TGF-β (R&D Systems) for 24 h. The methods for the production and infection of shRNA-expressing lentiviruses and cDNA-expressing retroviruses or the transfection of cDNA expression plasmids were essentially the same as described previously (43, 45).

### QRT–PCR

Total RNA was extracted with RNAiso plus (catalog no.: 9108; Takara) using a standard method and transcribed to cDNA using SuperScript Vilo cDNA synthesis kit (Invitrogen). QRT–PCR was performed as described previously (45). PCR data were normalized with control human *GAPDH* expression. The averages from at least three independent experiments are shown with the standard deviations. *p* Values were calculated between the control and the samples using Student’s *t* test. Primers used for the quantitative PCR were described previously (43, 45, 46) and are listed in Table S1.

### Immunoblotting and m6A-RIP

For immunoblotting, cells were lysed in radioimmunoprecipitation buffer as described previously (9). The lysates were separated on SuperSep Ace 10% running gel (Wako) and transferred to Hybond-LFP membrane (GE Healthcare). The antibodies used in this study include anti-JUN (catalog no.: 60A85; Cell Signaling Tech), anti-JUNB (catalog no.: C37F9; Cell Signaling Tech), anti-JUND (D-9; Santa Cruz Bio), anti-E-cadherin (catalog no.: 610181; BD Bioscience), anti-Vimentin (catalog no.: ab8069; Abcam), antifibronectin (catalog no.: SAB4500974; Sigma), anti-N-cadherin (catalog no.: 610921; BD Bioscience), anti-HA (catalog no.: 014-21881; Wako), anti-YTHDF1 (catalog no.: 17479-1-AP; Proteintech), anti-YTHDF2 (catalog no.: 24744-1-AP; Proteintech), anti-YTHDF3 (catalog no.: 25537-1-AP; Proteintech), anti-IGF2BP1 (catalog no.: 22803-1-AP; Proteintech), anti-IGF2BP2 (catalog no.:11601-1-AP; Proteintech), anti-IGF2BP3 (catalog no.: RN009P; MBL), anti-YTHDC1 (catalog no.: 29441-1-AP; Proteintech), anti-YTHDC2 (catalog no.: 27779-1-AP; Proteintech), anti-ALKBH5 (catalog no.: 16837-1-AP; Proteintech), and anti-FTO (catalog no.: 27226-1-AP; Proteintech). As a control, anti-GAPDH antibody (6C5; Millipore) was used to show that the same amount of protein was loaded. To quantify the GAPDH protein accurately in the separate blot with anti-GAPDH antibody, we used 1/20th the amount of each sample loaded in a usual immunoblotting. For protein stability assay, the cells were treated with cycloheximide (catalog no.: 06741; Nakarai, Kyoto) at 50 μg/ml to block protein synthesis. After incubation at the indicated periods, cell lysates were prepared for immunoblotting.

m6A RIP was conducted according to the previously described protocol (21). Briefly, 100 μg of total RNA was incubated with anti-m6A antibody (catalog no.: ab151230; Abcam), in 500 μl of RIP buffer (50 mM Tris–Cl pH 7.5, 150 mM NaCl, 10 mM EDTA, and 0.5% Nonidet-P40) supplemented with protease inhibitors (catalog no.: 03969-21; Nakarai) and SUPERase-In (catalog no.: AM2694; Thermo Fisher). Then the immunocomplexes were recovered with Protein G-coupled Dynabeads (catalog no.: 10003D; Thermo Fisher). The precipitated RNAs were extracted with High Pure RNA Tissue Kit (catalog no.: 11828665001; Roche) by following the manufacturer’s instruction and were quantified by QRT–PCR.

### Luciferase reporter assay

A549 cells with or without *METTL3* knockdown were seeded in triplicate in 24-well plates, and the luciferase reporter plasmid containing 5′UTR, CDS, or 3′UTR of *JUNB* was transfected using PEI MAX (catalog no.: 24765; Polysciences). The activities of Firefly luciferase and Renilla luciferase were measured 24 h later with the Dual-Luciferase Reporter Assay System according to the manufacturer's instructions (catalog no.: E1910; Promega). The relative luciferase activity (F-luc/R-luc) was calculated for each assay. To examine the mRNA expression levels of Firefly and Renilla luciferase, QRT–PCR was performed using the primers for F-luc and R-luc (Table S1).

### RIP assay

RIP assay was conducted according to the previously described protocol (8). In brief, cells were crosslinked with 0.5% paraformaldehyde for 10 min, and the complexes were fragmented by Bioruptor II ultrasonicator (BM Equipment Co). The crosslinked cell lysates were lysed with RIP buffer supplemented with protease inhibitors and SUPERase-In. The lysates were treated with anti-FLAG antibody (catalog no.: 018-22381; Wako) or normal mouse IgG bound to Dynabeads M-280 sheep antimouse IgG (Invitrogen). The coprecipitated RNAs were extracted with High Pure RNA Tissue Kit and were quantified by QRT–PCR. Percentage enrichment over input was presented.

### Statistical analysis

Unpaired Student’s *t* test was used to assess the statistical significance of the difference between the two groups. ∗∗*p* < 0.01; ∗*p* < 0.05; ns, not significant.

### Data availability

All data are included within the article and supporting information. The materials and methods in this study are available from the corresponding author upon request.

## Supporting information

This article contains supporting information.

## Conflict of interest

The authors declare that they have no conflicts of interest with the contents of this article.
