# Supplementary material for: m6A RNA methylation regulates the transcription factors JUN and JUNB in TGF-β-induced epithelial–mesenchymal transition of lung cancer cells
Source: J Biol Chem. 2022 Sep 29;298(11):102554. doi: 10.1016/j.jbc.2022.102554 (PMC9619186; doi:10.1016/j.jbc.2022.102554)
Supplement: Supplementary Information [file mmc1.docx]

**Supporting information (SI)**

**Title: m6A RNA methylation regulates the transcription factors JUN and JUNB in TGF-β-induced epithelial-mesenchymal transition of lung cancer cells**

**Suphakhong K. et al.**

1. **Supplementary Table S1**

**2. Supplementary Figure S1**

**3. Supplementary Figure S2**

**4. Supplementary Figure S3**

**5. Supplementary Figure S4**

**6. Supplementary Figure S5**

**7. Supplementary Figure S6**

**8. Supplementary Figure S7**

**9. Supplementary Figure S8**

**10. Supplementary Figure S9**

**Supplementary Table S1**

The sequences of oligonucleotides for quantitative PCR, shRNA-expression, cDNA cloning and mutant construction used in this study. The mutation sites were underlined.

| **Name** | **Primer sequence (5’ to 3’ )** |
| --- | --- |
| *JUN*  *qPCR* | F: CGCCTGATAATCCAGTCCA  R: TTCTTGGGGCACAGGAACT |
| *JUNB*  *qPCR* | F: CAAGGTGAAGACGCTCAAGG  R: TCATGACCTTCTGTTTGAGCTG |
| *JUND*  *qPCR* | F: CACAGTTCCTCTACCCCAAGG  R: TTCTGCTTGTGTAAATCCTCCA |
| *JUN*  *shRNA#1* | F: CCGGCGCAAACCTCAGCAACTTCAACTCGAGTTGAAGTTGCTGAGGTTTGCGTTTTTG  R: AATTCAAAAACGCAAACCTCAGCAACTTCAACTCGAGTTGAAGTTGCTGAGGTTTGCG |
| *JUN*  *shRNA#2* | F: CCGGCCTGATAATCCAGTCCAGCAACTCGAGTTGCTGGACTGGATTATCAGGTTTTTG  R: AATTCAAAAACCTGATAATCCAGTCCAGCAACTCGAGTTGCTGGACTGGATTATCAGG |
| *JUNB*  *shRNA#1* | F: CCGGCCCATCAACATGGAAGACCAACTCGAGTTGGTCTTCCATGTTGATGGGTTTTT  R: AATTAAAAACCCATCAACATGGAAGACCAACTCGAGTTGGTCTTCCATGTTGATGGG |
| *JUNB*  *shRNA#2* | F: CCGGTCATACACAGCTACGGGATACCTCGAGGTATCCCGTAGCTGTGTATGATTTTTG  R: AATTCAAAAATCATACACAGCTACGGGATACCTCGAGGTATCCCGTAGCTGTGTATGA |
| *JUND*  *shRNA#1* | F: CCGGCAAGAGTCAGAACACGGAGCTCTCGAGAGCTCCGTGTTCTGACTCTTGTTTTTG  R: AATTCAAAAACAAGAGTCAGAACACGGAGCTCTCGAGAGCTCCGTGTTCTGACTCTTG |
| *JUND*  *shRNA#2* | F: CCGGACGAGCTCACAGTTCCTCTACCTCGAGGTAGAGGAACTGTGAGCTCGTTTTTTG  R: AATTCAAAAAACGAGCTCACAGTTCCTCTACCTCGAGGTAGAGGAACTGTGAGCTCGT |
| *5’UTR_JUNB*  *pmirGLO* | F: GCTAGCGGGACCTTGAGAGCGGCCAG  R: CTCGAGCCGGGCGGCCCAGGC |
| *JUNB (cds)*  *pmirGLO* | F: GCTAGCATGTGCACTAAAATGGAACAGC  R: CTCGAGTCAGAAGGCGTGTCCCTTG |
| *JUNB_3’UTR*  *pmirGLO* | F: GCTAGCACGTCCCCTGCCCCTTTA  R: CTCGAGTTAATAAAGAACCAATTTTTTTTTT |
| *JUNB*  *RIP#1* | F: CAAGGGACACGCCTTCTGAA  R: CGTGTGCCCAGCCGTCCAAG |
| *JUNB*  *RIP#2* | F: GTCCAGGGAGCAGGCGGT  R: CAGGGTAGGAGGGCCGGAG |
| *JUNB*  *RIP#3* | F: TTTTTTTTCTGCTGGAAACAG  R: CCTCTTCCCCTCCCTGTTA |
| *JUNB m6A m1 mutant* | F: TTTACGG**G**CACCCCCTCGCTTGGACGG  R: GGGGGTG**C**CCGTAAAGGGGCAGGGGAC |
| *JUNB m6A m2 mutant* | F: CCCTGGG**G**CCTAGGGGCGCCGCAAACC  R: CCCTAGG**C**CCCAGGGTGGGTGCCCACC |
| *JUNB m6A m3 mutant* | F: ACACTGG**G**CTCCGGCCCTCCTACCCTG  R: GCCGGAG**C**CCAGTGTGGTTTGCGGCGC |
| *5’UTR_JUNB*  *construction* | F: GGATCCGGGACCTTGAGAGC  R: TCTAGAGAAGGCGTGTCCCTTGA |
| *JUNB (cds)*  *construction* | F: ACCCTCCCCAGACCGCCT  R: CCGTAAAGGGGCAGGGGA |
| *JUNB_3’UTR*  *construction* | F: TCTAGAATGTGCACTAAAATGGAA  R: AAGCTTTTAATAAAGAACCAATTTT |
| *F-luc*  *qPCR* | F: TGCAAAAGATCCTCAACGTG  R: GAAGCCCTGGTAGTCGGTCT |
| *R-luc*  *qPCR* | F: CTACTATGATTCCGAGAAGCACG  R: ACGACGTGCCTCCACAGGTA |
| *5'UTR_JUNB/HA*  *qPCR* | F: ACGTCAGCAACGGCTGTCAG  R: AGCGTAGTCTGGGACGTCGTA |
| *HA/JUNB_3'UTR*  *qPCR* | F: TATCCTTATGACGTGCCTGACTAT  R: TGTATGAGTCGTCGTGGTAGAAG |
| *5’UTR_JUN*  *pmirGLO* | F: GCTAGCGTTGACAGCGGCGGAAAG  R: CTCGAGGAGGGCATCGTCATAGAAGG |
| *JUN (cds)*  *pmirGLO* | F: GCTAGCATGACTGCAAAGATGGAAACG  R: CTCGAGTCAAAATGTTTGCAACTGCTG |
| *JUN_3’UTR*  *pmirGLO* | F: GCTAGCGGCTGGAGGAAAAAGTGAAA  R: CTCGAGGGCAGGATACCCAAACAAAC |
| *5’UTR_JUN*  *construction* | F: TTCTCCACGGCGGTAAAG  R: TCAAAATGTTTGCAACTGCTG |
| *JUN (cds)*  *construction* | F: CGCTCTTAGAGAAACTTTCCCTGT  R: CTCTGTGTTATTTTTTTTCTTCGTTG |
| *JUN_3’UTR*  *construction* | F: ATGACTGCAAAGATGGAAACGA  R: CCCCCGCTTTGTGTTCTTA |
| *5’UTR_JUN_3’UTR*  *construction* | F: CCATCACCATTGAGCAGAGAGACCGTCGGGG  R: TAACGTCGAGCGGCCTTGGTATTTGAATACATTTATTGTG |
| *JUN*  *RIP#1* | F: AACAGAAAGTCATGAACCACGTT  R: CCGACGGTCTCTCTTCAAAA |
| *JUN*  *RIP#2* | F: GAGGGGCAACGAAGAAAAA  R: CTTGGCTTTAGTTCTCGGACA |
| *JUN*  *RIP#3* | F: GAACGGAACGTTGGACTTTT  R: CCCCTTTAATACTGAATGAGATCG |
| *JUN*  *RIP#4* | F: GGCTGAGCCTACAGATGAACTC  R: CAGTAATCAGCTTTCATCAAATTAAAA |
| *JUN m6A m1*  *mutant* | F: AAGAGAG**G**CCGTCGGGGGCTGAGGGGC  R: CCGACGG**C**CTCTCTTCAAAATGTTTGCA |
| *JUN m6A m2*  *mutant* | F: AAAAATA**G**CACAGAGAGACAGACTTGA  R: CTCTGTG**C**TATTTTTTTTCTTCGTTGC |
| *JUN m6A m3*  *mutant* | F: CTTGAGA**G**CTTGACAAGTTGCGACGGA  R: TGTCAAG**C**TCTCAAGTCTGTCTCTCTG |
| *5'UTR_CJUN/HA*  *qPCR* | F: CTCAGGGAACAGGTGGCACA  R: AGCGTAGTCTGGGACGTCGTA |
| *HA/CJUN_3'UTR*  *qPCR* | F: TATCCTTATGACGTGCCTGACTAT  R: ATCTTGGGGTTACTGTAGCCATAA |
| *YTHDF1*  *shRNA#1* | F: CCGGCCCGAAAGAGTTTGAGTGGAACTCGAGTTCCACTCAAACTCTTTCGGGTTTTTG  R: AATTCAAAAACCCGAAAGAGTTTGAGTGGAACTCGAGTTCCACTCAAACTCTTTCGGG |
| *YTHDF1*  *shRNA#2* | F: CCGGCGGTGGGACAAATGTGAACATCTCGAGATGTTCACATTTGTCCCACCGTTTTTG  R: AATTCAAAAACGGTGGGACAAATGTGAACATCTCGAGATGTTCACATTTGTCCCACCG |
| *YTHDF2*  *shRNA#1* | F: CCGGGACTTCTCACACTATGAGAAACTCGAGTTTCTCATAGTGTGAGAAGTCTTTTTTG  R: AATTCAAAAAAGACTTCTCACACTATGAGAAACTCGAGTTTCTCATAGTGTGAGAAGTC |
| *YTHDF2*  *shRNA#2* | F: CCGGTCTGGATATAGTAGCAATTATCTCGAGATAATTGCTACTATATCCAGATTTTTG  R: AATTCAAAAATCTGGATATAGTAGCAATTATCTCGAGATAATTGCTACTATATCCAGA |
| *YTHDF3*  *shRNA#1* | F: CCGGTAAGTCAAAGAAGACGTATTACTCGAGTAATACGTCTTCTTTGACTTATTTTTG  R: AATTCAAAAATAAGTCAAAGAAGACGTATTACTCGAGTAATACGTCTTCTTTGACTTA |
| *YTHDF3*  *shRNA#2* | F: CCGGGATAAGTGGAAGGGCAAATTTCTCGAGAAATTTGCCCTTCCACTTATCTTTTTG  R: AATTCAAAAAGATAAGTGGAAGGGCAAATTTCTCGAGAAATTTGCCCTTCCACTTATC |
| *IGF2BP1*  *shRNA#1* | F: CCGGACGCTTAGAGATTGAACATTCCTCGAGGAATGTTCAATCTCTAAGCGTTTTTTTG  R: AATTCAAAAAAACGCTTAGAGATTGAACATTCCTCGAGGAATGTTCAATCTCTAAGCGT |
| *IGF2BP1*  *shRNA#2* | F: CCGGGATGGATGCTACGAGTATAAACTCGAGTTTATACTCGTAGCATCCATCTTTTTG  R: AATTCAAAAAGATGGATGCTACGAGTATAAACTCGAGTTTATACTCGTAGCATCCATC |
| *IGF2BP2*  *shRNA#1* | F: CCGGGCCGTTGTCAACGTCACATATCTCGAGATATGTGACGTTGACAACGGCTTTTTG  R: AATTCAAAAAGCCGTTGTCAACGTCACATATCTCGAGATATGTGACGTTGACAACGGC |
| *IGF2BP2*  *shRNA#2* | F: CCGGAGCGCAAGATCAGGGAAATTGCTCGAGCAATTTCCCTGATCTTGCGCTTTTTTG  R: AATTCAAAAAAGCGCAAGATCAGGGAAATTGCTCGAGCAATTTCCCTGATCTTGCGCT |
| *IGF2BP3*  *shRNA#1* | F: CCGGTGTTGTAGTCTCACAGTATAACTCGAGTTATACTGTGAGACTACAACATTTTTG  R: AATTCAAAAATGTTGTAGTCTCACAGTATAACTCGAGTTATACTGTGAGACTACAACA |
| *IGF2BP3*  *shRNA#2* | F: CCGGGCCTCATTCTTATTTCAAGATCTCGAGATCTTGAAATAAGAATGAGGCTTTTTG  R: AATTCAAAAAGCCTCATTCTTATTTCAAGATCTCGAGATCTTGAAATAAGAATGAGGC |
| *YTHDC1*  *shRNA#1* | F: CCGGTGCCTCCAGAGAACCTTATAACTCGAGTTATAAGGTTCTCTGGAGGCATTTTTG  R: AATTCAAAAATGCCTCCAGAGAACCTTATAACTCGAGTTATAAGGTTCTCTGGAGGCA |
| *YTHDC1*  *shRNA#2* | F: CCGGTGGATTTGCAGGCGTGAATTACTCGAGTAATTCACGCCTGCAAATCCATTTTTG  R: AATTCAAAAATGGATTTGCAGGCGTGAATTACTCGAGTAATTCACGCCTGCAAATCCA |
| *YTHDC2*  *shRNA#1* | F: CCGGCGGAAGCTAAATCGAGCCTTTCTCGAGAAAGGCTCGATTTAGCTTCCGTTTTTG  R: AATTCAAAAACGGAAGCTAAATCGAGCCTTTCTCGAGAAAGGCTCGATTTAGCTTCCG |
| *YTHDC2*  *shRNA#2* | F: CCGGGCTCAGGTCTTTCATCTCATTCTCGAGAATGAGATGAAAGACCTGAGCTTTTTG  R: AATTCAAAAAGCTCAGGTCTTTCATCTCATTCTCGAGAATGAGATGAAAGACCTGAGC |
| *YTHDF1*  *qPCR* | F: CGACGACTTTGCTCACTACG  R: TTCGACTCTGCCGTTCCTT |
| *YTHDF2*  *qPCR* | F: GGCAGCACTGAAGTTGGG  R: CTATTGGAAGCCACGATGTTA |
| *YTHDF3*  *qPCR* | F: CTCAACCACCACAACCACAG  R: CTGCTGTTGAGGCTGCACT |
| *IGF2BP1*  *qPCR* | F: CCTGCTGGCTCAGTATGGT  R: GACATTCACCACTGCCGTCTC |
| *IGF2BP2*  *qPCR* | F: CGGATACTTCTCCAGCCTGT  R: TCACAATCTCCTGCTCTGGAT |
| *IGF2BP3*  *qPCR* | F: TGGAGCCATCATAGGAAAAGA  R: CGCATTTTCTTTACGGTGGA |
| *YTHDC1*  *qPCR* | F: TGTGAGGGAATTTCATAACATGG  R: GGTTGTTCCATTCCTGGGTA |
| *YTHDC2*  *qPCR* | F: TGACTGATGAGTATGACTTACTGGATG  R: CAATTCACATCTTTTTCAGTCAGC |
| *YTHDF3*  *construction* | F: CTCATTTTGGGTTCTCAGCG  R: CATTACGAGAAGAGTCAACTTTGTG |
| *IGF2BP1*  *Construction* | F: CTGCCCCGAGACCGCCAC  R: GATTTCTGCCCGTTGTTGTCCTG |
| *ALKBH5*  *qPCR* | F: CCTGCTCTGAAACCCAAGC  R: TCCTTGTCCATCTCCAGGAT |
| *FTO*  *qPCR* | F: GAAAATCTGGTGGACAGGTCA  R: CGAGATGAGAGTCATCCTCACTT |

F: Forward, R: Reverse


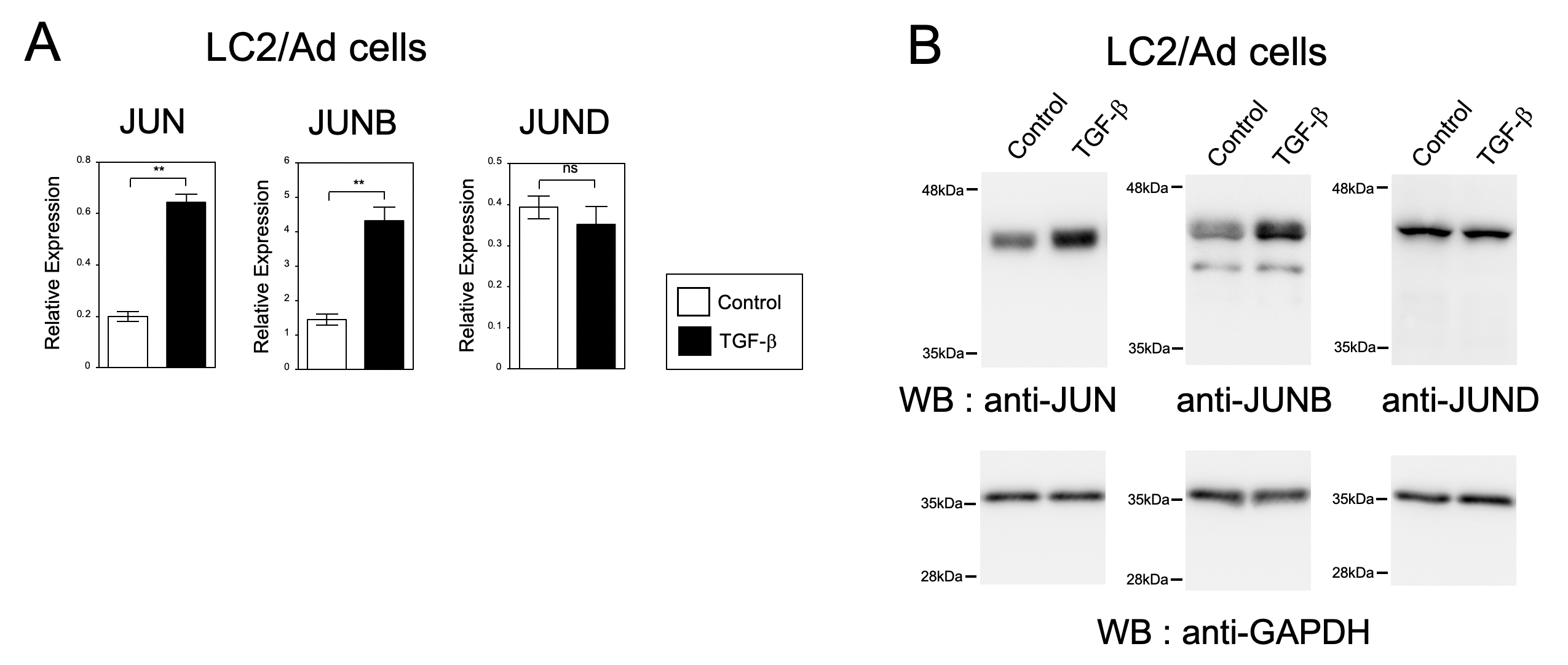


**Supplementary Fig. S1.** The expression of JUN family mRNAs and proteins in LC2/ad lung cancer cells

(A) The expression of *JUN*, *JUNB* and *JUND* mRNAs in TGF-β-induced EMT of LC2/ad cells. QRT-PCR was performed in LC2/ad cells with or without TGF-β treatment (**, *P* < 0.01; ns, not significant). (B) The expression levels of JUN family proteins in LC2/ad cells. Immunoblotting was performed to detect JUN, JUNB and JUND proteins in the cells with or without TGF-β treatment. As a control, anti-GAPDH antibody was used to show that equal amounts of proteins were loaded.


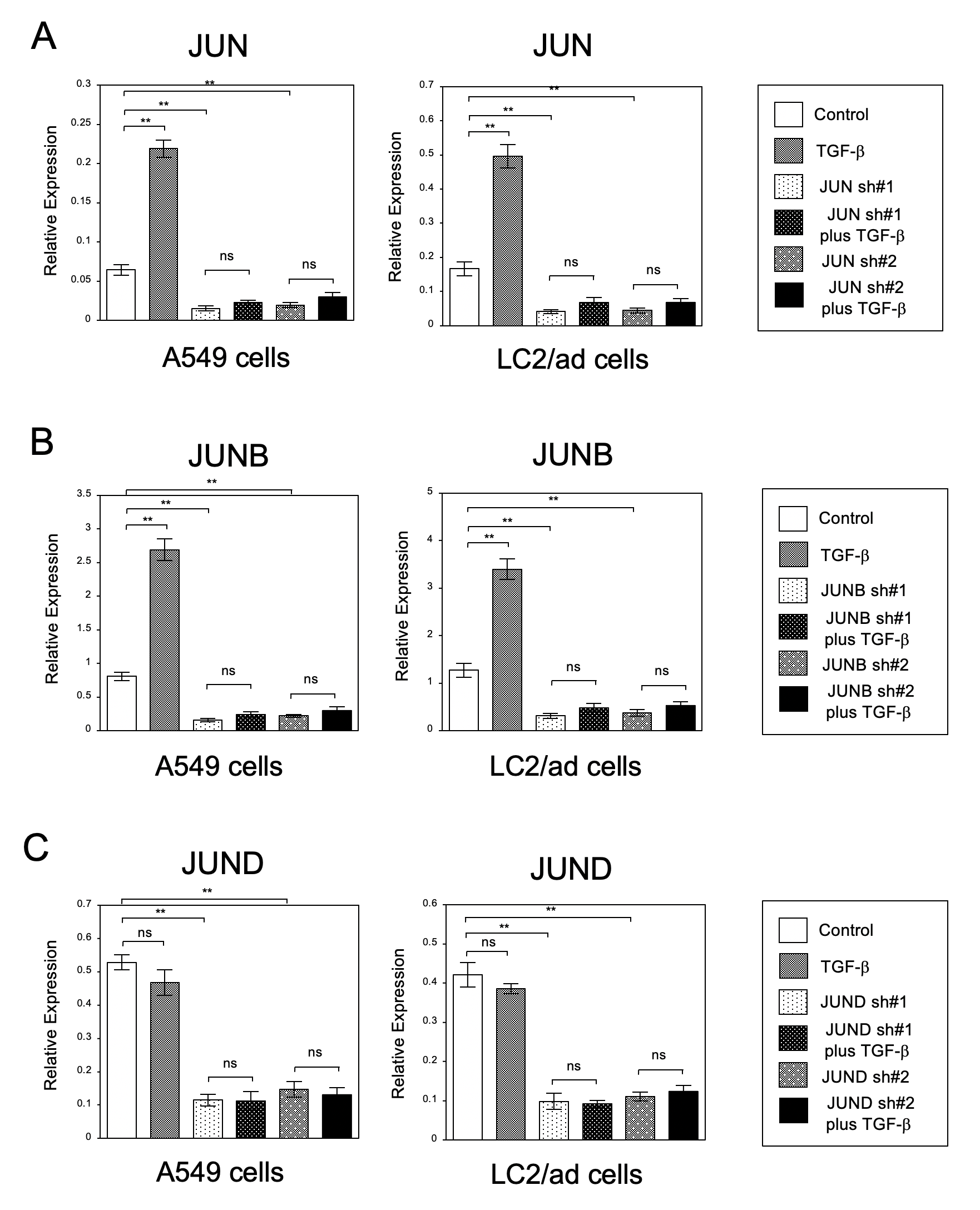


**Supplementary Fig. S2.** Knockdown efficiencies of JUN family members by their shRNAs in A549 and LC2/ad cells.

(A) QRT-PCR was performed to detect the expression of *JUN* in A549 or LC2/ad cells infected with the lentivirus expressing control shRNA, *JUN* shRNA#1 or *JUN* shRNA#2 without or with TGF-β for 24 hours (n=3) (**, *P* < 0.01; ns, not significant). Similar QRT-PCR results are shown for the knockdown of *JUNB* (B) and *JUND* (C) in both cells.


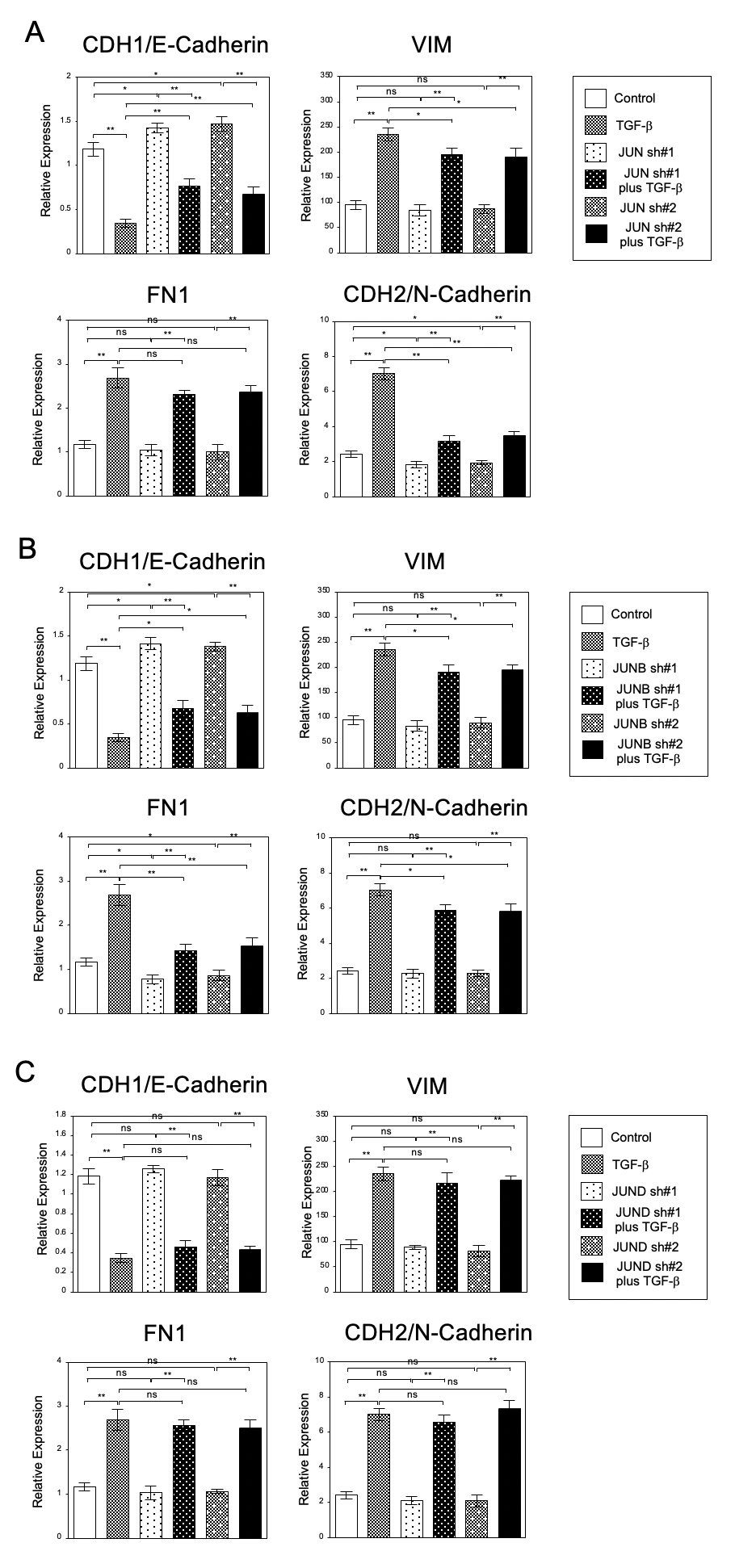


**Supplementary Fig. S3.** Knockdown of *JUN* and *JUNB* but not of *JUND* affected the mRNA expression changes of EMT-related marker genes induced by TGF-β in A549 cells.

(A) The expression of EMT-related marker genes in the *JUN* knockdown cells. QRT-PCR was performed to detect the expression of *CDH1*, *VIM*, *FN1* and *CDH2* in A549 cells infected with the lentivirus expressing control shRNA, *JUN* shRNA#1 or *JUN* shRNA#2 without or with TGF-β for 24 hours (n=3) (**, *P* < 0.01; *, *P* < 0.05; ns, not significant). Similar QRT-PCR results are shown for the knockdown of *JUNB* (B) and *JUND* (C) in A549 cells.

**
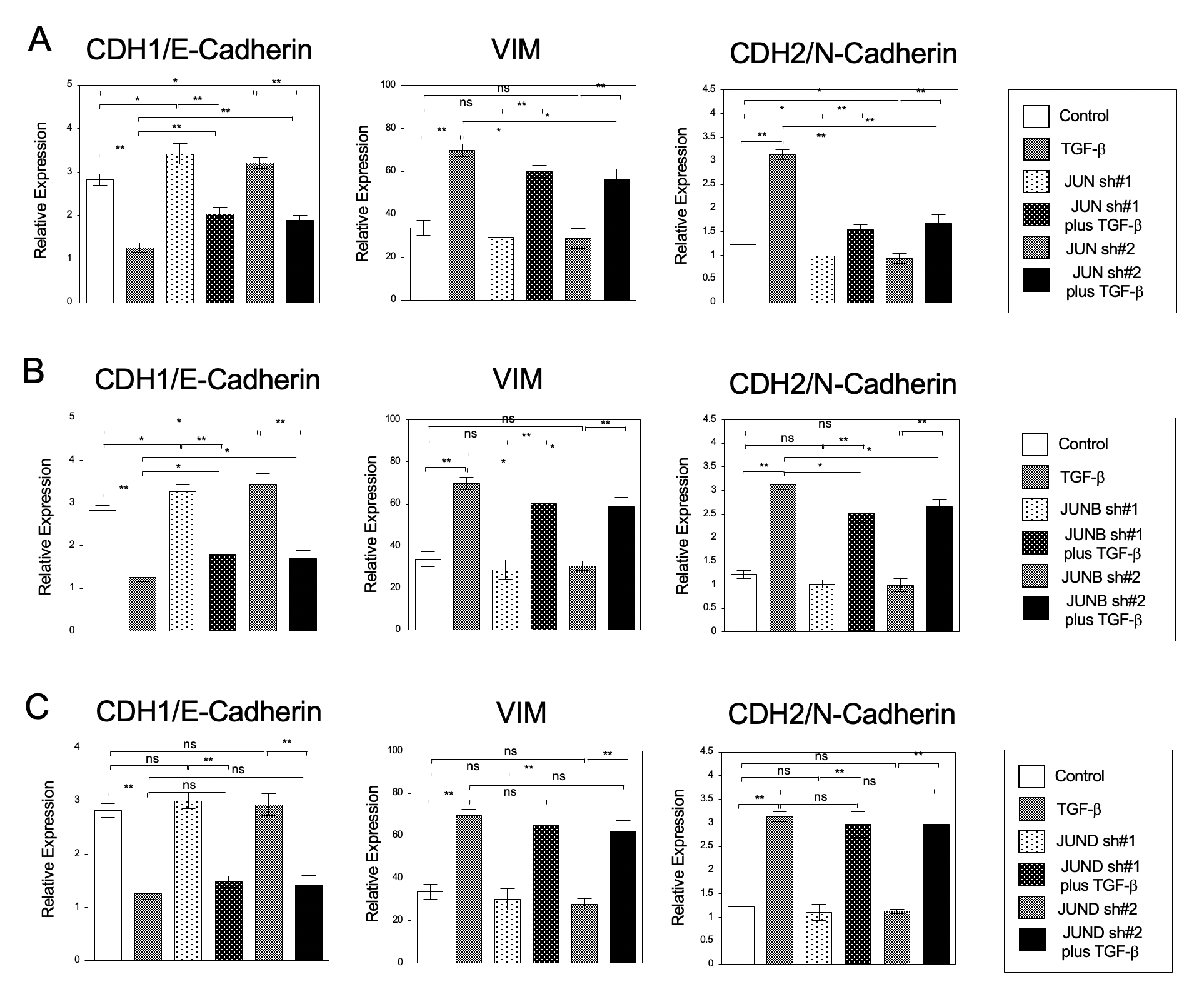
**

**Supplementary Fig. S4.** Knockdown of *JUN* and *JUNB* but not of *JUND* affected the mRNA expression changes of EMT-related marker genes induced by TGF-β in LC2/ad cells.

(A) The expression of EMT-related marker genes in the *JUN* knockdown cells. QRT-PCR was performed to detect the expression of *CDH1*, *VIM* and *CDH2* in LC2/ad cells infected with the lentivirus expressing control shRNA, *JUN* shRNA#1 or *JUN* shRNA#2 without or with TGF-β for 24 hours (n=3) (**, *P* < 0.01; *, *P* < 0.05; ns, not significant). *FN1* expression was too low to be analyzed in LC2/ad cells. Similar QRT-PCR results are shown for the knockdown of *JUNB* (B) and *JUND* (C) in LC2/ad cells.


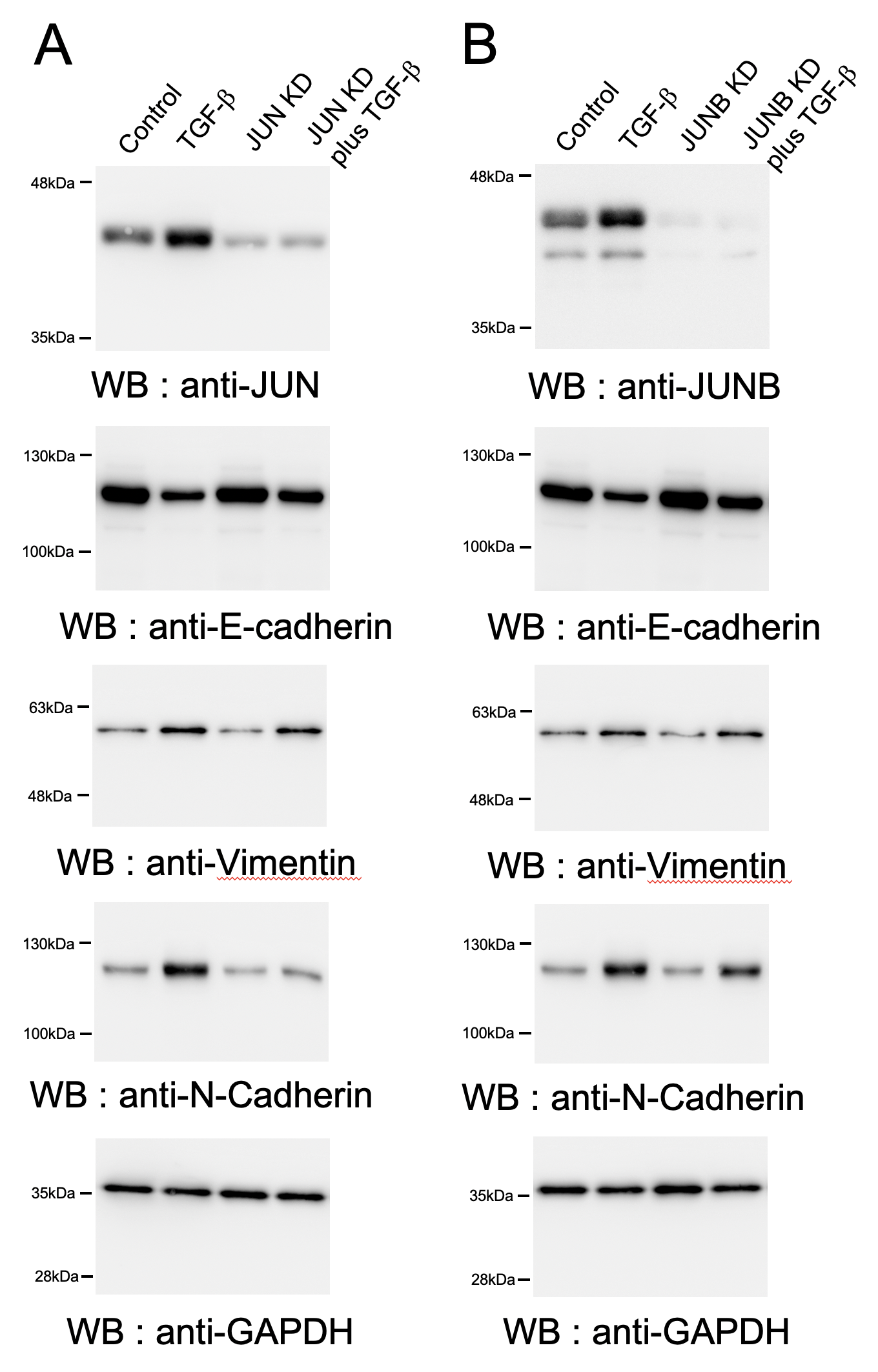


**Supplementary Fig. S5.** Knockdown of *JUN* and *JUNB* affected the protein expression changes of EMT-related marker genes induced by TGF-β in LC2/ad cells.

(A)(B) Immunoblotting of JUN, JUNB, E-cadherin, Vimentin, N-cadherin and GAPDH proteins was performed using the corresponding antibodies in LC2/ad cells with the knockdown by *JUN* shRNA#1 (A) and *JUNB* shRNA#1 (B).


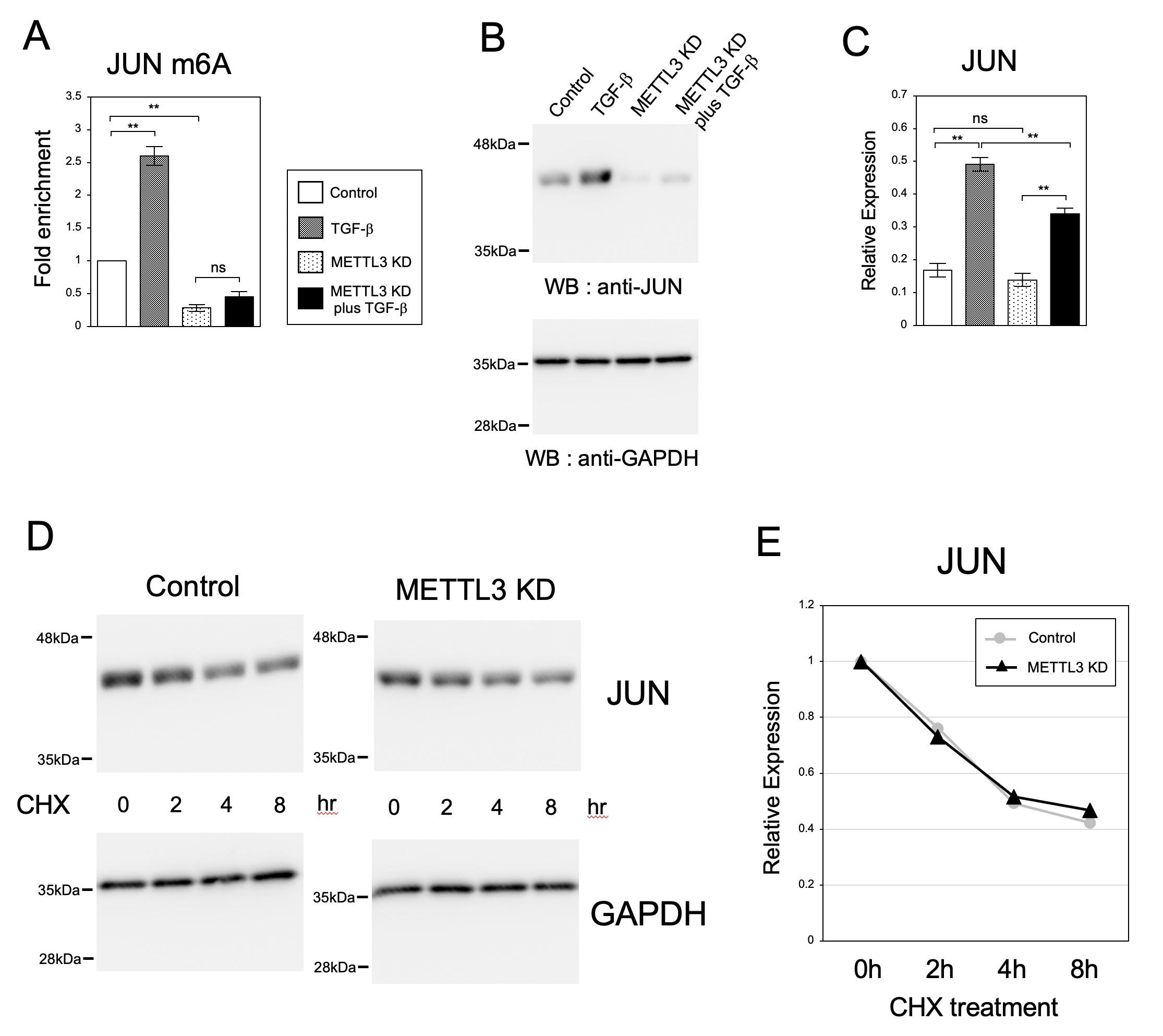


**Supplementary Fig. S6.** Knockdown of *METTL3* decreased the m6A modification of *JUN* mRNA and the expression of JUN protein but did not affect the stability of JUN protein in LC2/ad cells.

(A) The m6A methylated *JUN* mRNA level in the *METTL3* knockdown LC2/ad cells. The m6A-IP-QPCR assay was performed in the control or *METTL3* knockdown LC2/ad cells with or without TGF-β for 24 hours (n=3) (**, *P* < 0.01; ns, not significant). (B) The JUN protein level in the *METTL3* knockdown cells. Immunoblotting was performed to detect JUN protein in the cells shown in (A). As a control, anti-GAPDH antibody was used. (C) The total *JUN* mRNA level in the *METTL3* knockdown cells. QRT-PCR for *JUN* was performed in the cells shown in (A) (n=3) (**, *P* < 0.01; ns, not significant). (D)(E) The protein stability of JUN in the *METTL3* knockdown cells. The control and *METTL3* knockdown LC2/ad cells were treated with cycloheximide (CHX) for the indicated times, and protein expression of JUN was analyzed by immunoblotting (D). The band intensities were measured, and the quantitative values of JUN protein normalized by GAPDH expression were plotted (E).


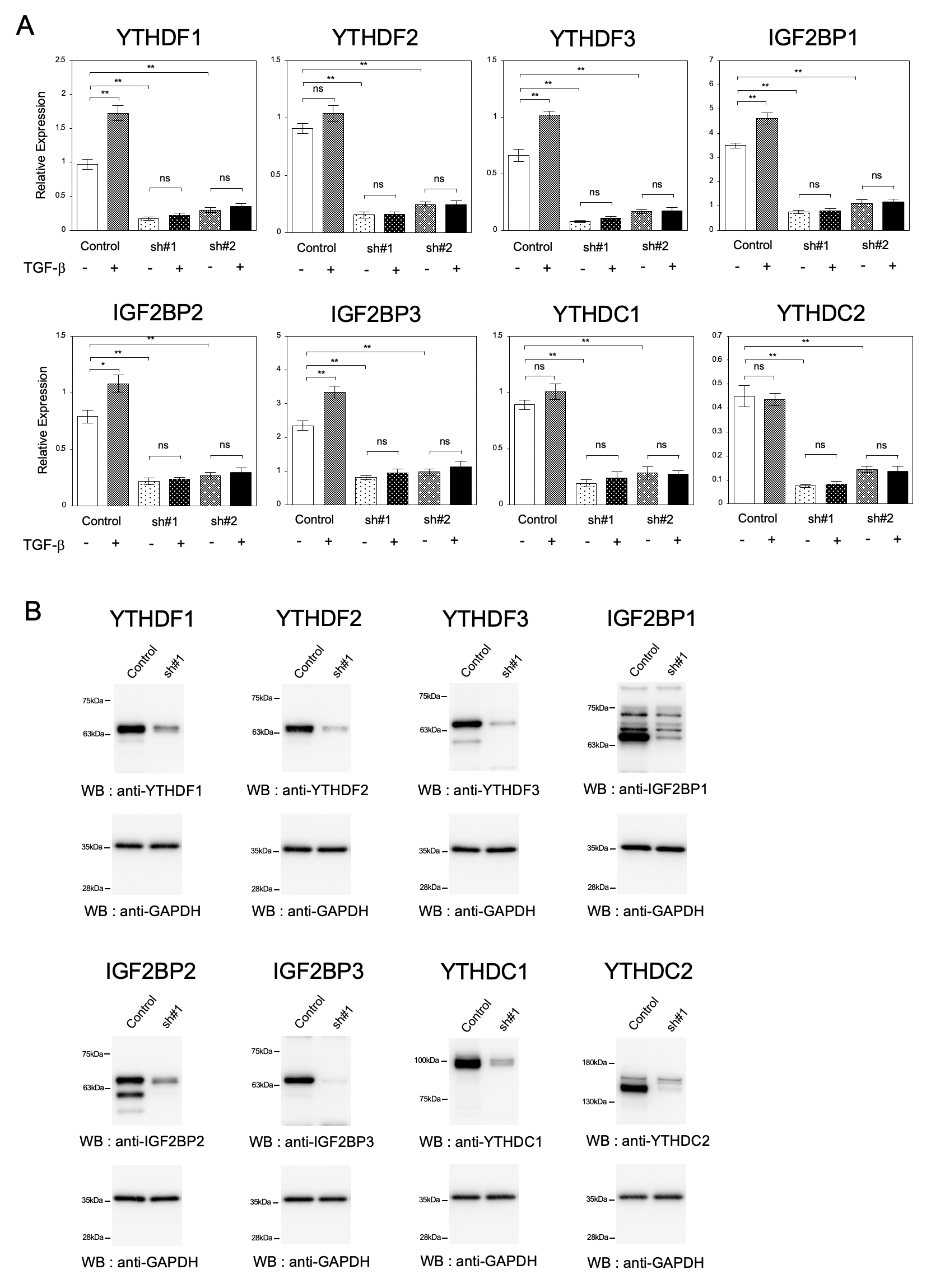


**Supplementary Fig. S7.** Knockdown efficiencies of the m6A reader proteins by the corresponding shRNAs in A549 cells.

(A) QRT-PCR was performed to detect the expression of each m6A reader in A549 cells infected with the lentivirus expressing control shRNA, the corresponding shRNA#1 or shRNA #2 of each m6A reader without or with TGF-β treatment for 24 hours (n=3) (**, *P* < 0.01; *, *P* < 0.05; ns, not significant). (B) Immunoblotting of each m6A reader protein was performed using the corresponding antibody in A549 cells with the knockdown by control shRNA and the corresponding shRNA#1. As a loading control, anti-GAPDH antibody was used.


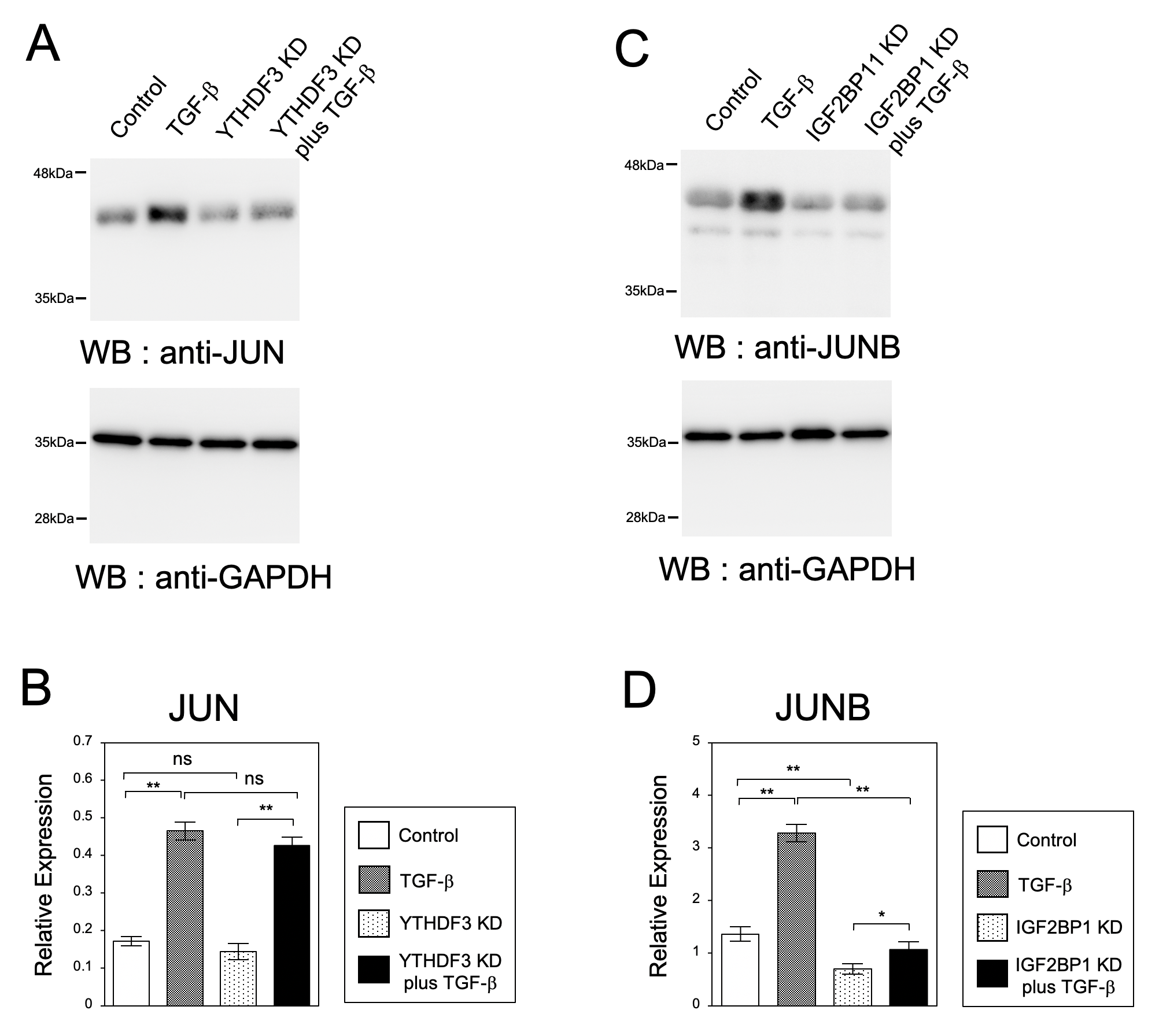


**Supplementary Fig. S8.** Knockdown of *YTHDF3* and *IGF2BP1* affected the expression of JUN and JUNB, respectively, in LC2/ad cells.

(A)(B) The expression of JUN protein (A) and *JUN* mRNA (B) in the *YTHDF3* knockdown cells. Immunoblotting was performed to detect JUN protein in the control or *YTHDF3* knockdown LC2/ad cells with or without TGF-β (A). As a control, anti-GAPDH antibody was used. QRT-PCR for *JUN* was performed in the same set of cells (B) (n=3) (**, *P* < 0.01; *, *P* < 0.05; ns, not significant). (C)(D) The expression of JUNB protein (C) and *JUNB* mRNA (D) in the *IGF2BP1* knockdown LC2/ad cells.


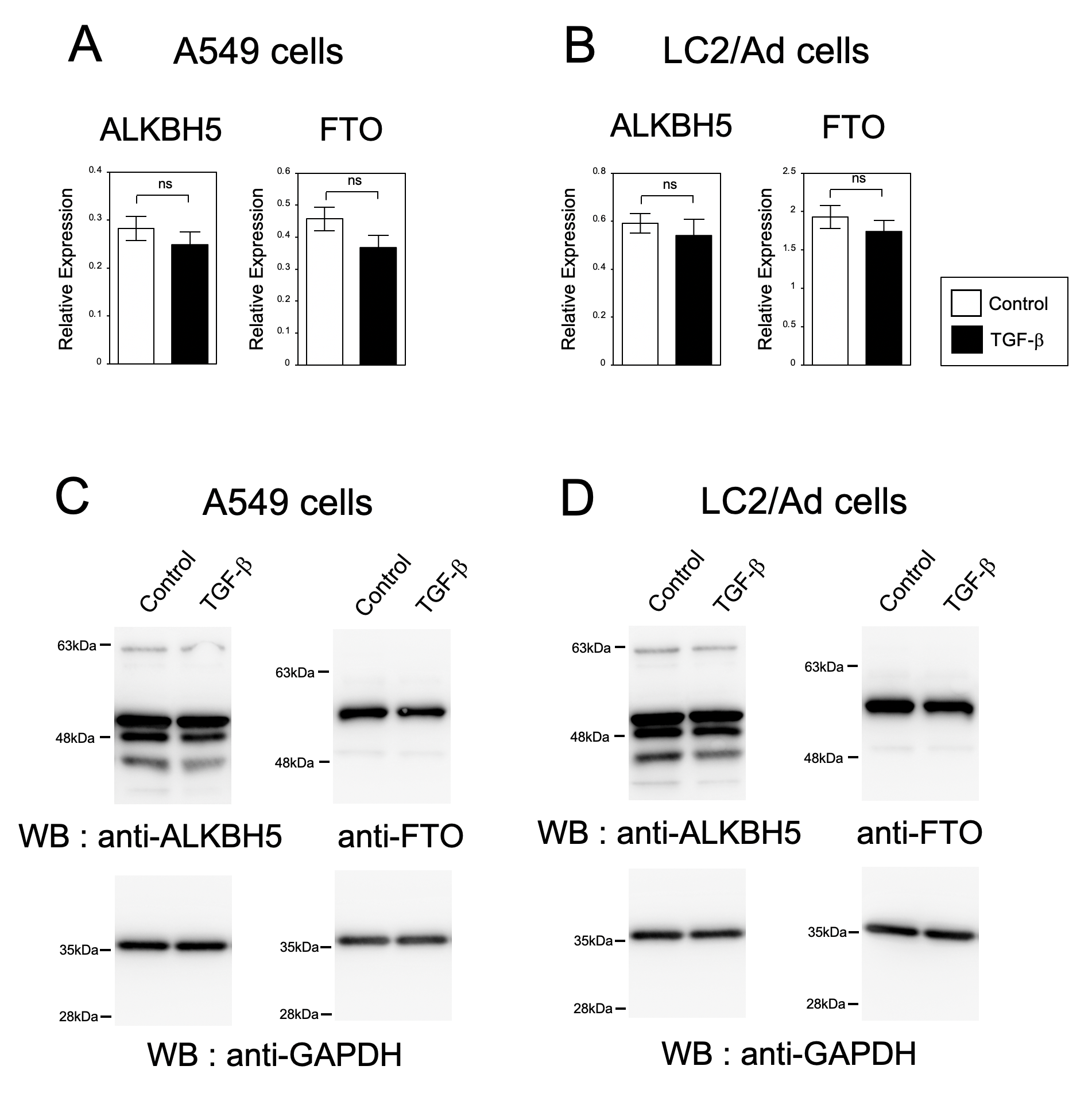


**Supplementary Fig. S9.** The expression of m6A demethylases, ALKBH5 and FTO, in TGF-β-induced EMT of A549 and LC2/ad lung cancer cells

(A)(B) The expression of *ALKBH5* and *FTO* mRNAs in TGF-β-induced EMT of A549 and LC2/ad cells. QRT-PCR was performed in A549 (A) and LC2/ad (B) cells with or without TGF-β treatment (ns, not significant). (C)(D) The expression levels of ALKBH5 and FTO proteins in A549 and LC2/ad cells. Immunoblotting was performed to detect ALKBH5 and FTO proteins in A549 (C) and LC2/ad (D) cells with or without TGF-β treatment. The anti-ALKBH5 antibody detects several isoforms of ALKBH5 proteins with molecular weights of 40-52 kDa according to the manufacturer’s information. As a control, anti-GAPDH antibody was used to show that equal amounts of proteins were loaded.
